# A nanoselenium-coating biomimetic cytomembrane nanoplatform for mitochondrial targeted chemotherapy- and chemodynamic therapy through manganese and doxorubicin codelivery

**DOI:** 10.1186/s12951-021-00971-9

**Published:** 2021-07-30

**Authors:** Jianmin Xiao, Miao Yan, Ke Zhou, Hui Chen, Zhaowei Xu, Yuehao Gan, Biao Hong, Geng Tian, Junchao Qian, Guilong Zhang, Zhengyan Wu

**Affiliations:** 1 Key Laboratory of High Magnetic Field and Ion Beam Physical Biology, Hefei Institutes of Physical Science, Chinese Academy of Sciences, Hefei, 230031 People’s Republic of China; 2 University of Science and Technology of China, Hefei, 230026 People’s Republic of China; 3 School of Pharmacy, The Key Laboratory of Prescription Effect and Clinical Evaluation of State Administration of Traditional Chinese Medicine of China, Binzhou Medical University, Yantai, 264003 People’s Republic of China; 4 Department of Dental Implant Center, Key Laboratory of Oral Diseases Research of Anhui Province, Stomatologic Hospital & College, Anhui Medical University, Hefei, 230032 People’s Republic of China; 5 Anhui Province Key Laboratory of Medical Physics and Technology, Institute of Health and Medical Technology, Hefei Institutes of Physical Science, Hefei Cancer Hospital, Chinese Academy of Sciences, Hefei, 230031 People’s Republic of China

**Keywords:** Cell membrane, Reactive oxygen species, Superoxide dismutase-1, Glutathione peroxidase 4, Dual-mode therapeutic pathways, Drug delivery system

## Abstract

The cell membrane is widely considered as a promising delivery nanocarrier due to its excellent properties. In this study, self-assembled *Pseudomonas geniculate* cell membranes were prepared with high yield as drug nanocarriers, and named BMMPs. BMMPs showed excellent biosafety, and could be more efficiently internalized by cancer cells than traditional red cell membrane nanocarriers, indicating that BMMPs could deliver more drug into cancer cells. Subsequently, the BMMPs were coated with nanoselenium (Se), and subsequently loaded with Mn^2+^ ions and doxorubicin (DOX) to fabricate a functional nanoplatform (BMMP-Mn^2+^/Se/DOX). Notably, in this nanoplatform, Se nanoparticles activated superoxide dismutase-1 (SOD-1) expression and subsequently up-regulated downstream H_2_O_2_ levels. Next, the released Mn^2+^ ions catalyzed H_2_O_2_ to highly toxic hydroxyl radicals (·OH), inducing mitochondrial damage. In addition, the BMMP-Mn^2+^/Se nanoplatform inhibited glutathione peroxidase 4 (GPX4) expression and further accelerated intracellular reactive oxygen species (ROS) generation. Notably, the BMMP-Mn^2+^/Se/DOX nanoplatform exhibited increased effectiveness in inducing cancer cell death through mitochondrial and nuclear targeting dual-mode therapeutic pathways and showed negligible toxicity to normal organs. Therefore, this nanoplatform may represent a promising drug delivery system for achieving a safe, effective, and accurate cancer therapeutic plan.

**Supplementary Information:**

The online version contains supplementary material available at 10.1186/s12951-021-00971-9.

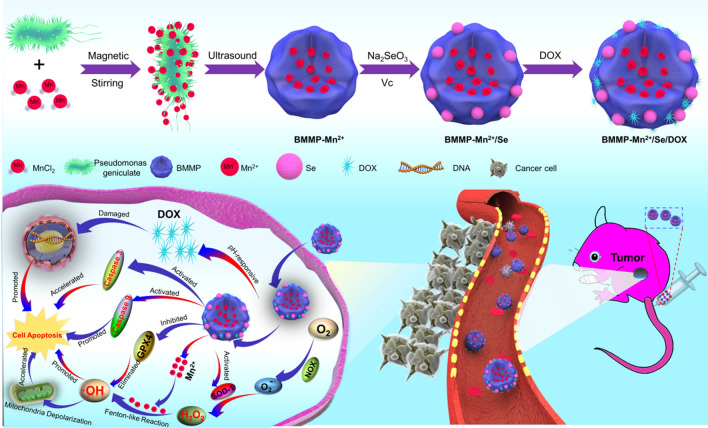

## Introduction

Drug delivery systems (DDSs) are actively exploited to enhance the delivery efficiency of drugs and decrease their systemic side effects [1–6]. The biosafety and simple fabrication of DDSs are critical factors for promoting their wide application in the biomedical field [7–9]. In recent decades, an increasing number of studies reported that some DDSs including metal-, silica-, carbon-, or polymer-based nanocarriers exhibited potential side effects [10–18], which significantly limited their clinical use. Currently, biomimetic nanomaterials (BMNs) have attracted extensive attention in worldwide and exhibit great application potential for DDSs due to their excellent biocompatibility, long blood circulation time, and internalization efficiency [19–22]. Among these, as a classical BMN, the cell membrane shows a unique advantage in effectively delivering bioactive molecules or anticancer drugs to the tumor site [23–27]. However, along with the wide application of the cell membrane in DDSs, some limitations have been gradually exposed, such as the complexity of preparation technology, uncontrollable morphology, and poor colloid stability, which seriously limit cell membrane-based DDSs in clinical use. Therefore, it is highly important to develop methods for the preparation of biomimetic cell membranes in a simple and high-yield manner.

In addition, for DDSs, the choice of chemotherapeutics is a key point to obtain an effective cancer therapy effect. Traditionally, to realize specific functions, DDSs typically load a single drug and can effectively deliver drugs to targeting regions [28–31]. However, the use of DDSs with a single drug easily induced the acquired multidrug resistance (MDR) of tumors [32–34]. To avoid these disadvantages, some studies reported that multiple chemotherapeutics were simultaneously loaded in DDSs, making suitable for the combined therapy with multiple drugs [35–37]. However, this strategy dramatically enhanced the dosage of chemotherapeutic drugs, which might increase the potential risk to the body. Therefore, the development of nanoplatforms that integrate chemotherapy with other therapy methods might provide a new breakthrough to improve MDR. Recently, as an alternative to traditional therapeutic modalities, chemodynamic therapy (CDT) has received more attention because CDT agents can generate abundant reactive oxygen species (ROS), which significantly induce mitochondrial damage in cancer cells [38–43]. Mitochondria that are widely distributed in the cytoplasm play a critical role in maintaining the normal growth of cells. Therefore, CDT can solve the MDR of cancer through the mitochondria-mediated cell death pathway [44]. Therefore, the integration of CDT and chemotherapy might provide a more effective pattern for improving the therapeutic efficacy of cancer.

**Scheme 1:**
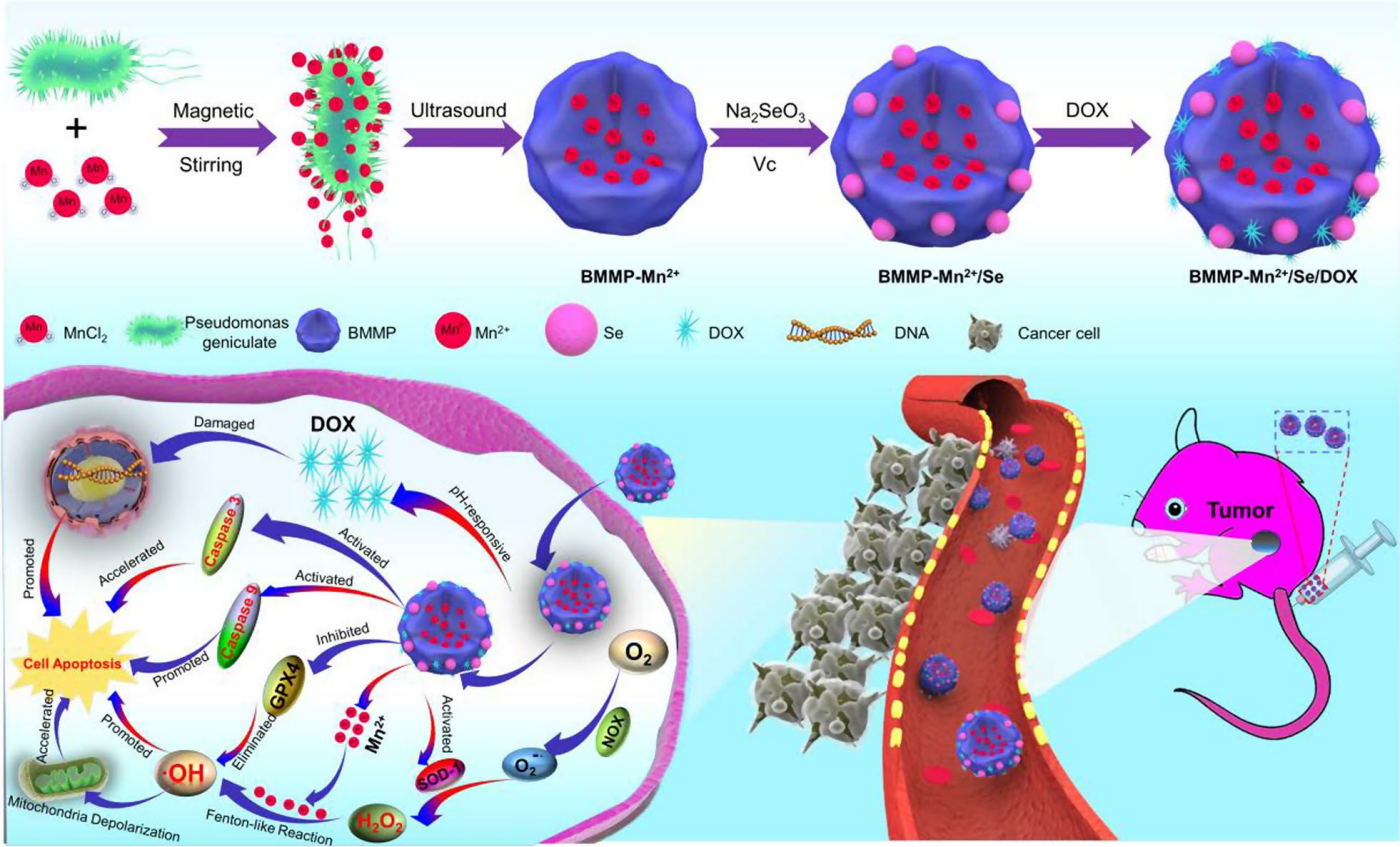
Illustration of the synthetic process of BMMP-Mn^2+^/Se/DOX and the nanoplatform integrating chemodynamic therapy and chemotherapeutics for cancer therapy

Herein, we prepared a biomimetic membrane (BMM) with high yield as a biocompatible nanocarrier to fabricate DDS through a simple ultrasonic treatment for *Pseudomonas geniculate* and named it BMMP. Compared to the carrier of traditional red cell membranes (RCMs), this nanoplatform exhibited more effective internalization efficiency by cancer cells, which would be beneficial to effectively deliver anticancer agents to tumor tissues. Subsequently, Mn^2+^ ions were absorbed into BMMP and then formed BMMP-Mn^2+^. Next, BMMP-Mn^2+^ was further used to load nanoselenium and DOX, forming a BMMP-Mn^2+^/Se/DOX nanoplatform for cancer therapy. Furthermore, when this nanoplatform entered cancer cells, it could enhance superoxide dismutase-1 (SOD-1) activity and then promote SOD-1 expression as illustrated in Scheme 1, accelerating the conversion of superoxide anion (O_2_^−·^) to hydrogen peroxide (H_2_O_2_). Next, the generated H_2_O_2_ could be catalyzed into hydroxyl radicals (·OH), causing damage to mitochondria and lipid peroxide and subsequently inducing cancer cell death. In addition, the BMMP-Mn^2+^/Se nanoplatform inhibited glutathione peroxidase 4 (GPX4) expression and further up-regulated intracellular ROS. Notably, after BMMP-Mn^2+^/Se treatment, the mitochondrial membrane potential (MMP) of cancer cells dramatically decreased, and some apoptotic enzymes (i.e., cleaved caspase-3/9) induced by mitochondria significantly increased, resulting in mitochondria-targeting cell death. In addition, the released DOX entered nuclear and then inhibited DNA replication, resulting in cancer cell death. Therefore, this work provides a safe, effective, and promising platform for chemotherapeutic- and chemodynamic combination cancer therapy.

## Materials and methods

### Materials

All chemical reagents were used as received without further purification. The *Pseudomonas geniculate* was isolated from wheat seed samples. Fluorescein isothiocyanate (FITC), 3′, 3′, 5′, 5′-tetramethylbenzidine (TMB), ascorbic acid (Vc), and DOX·HCl were obtained from Aladdin Chemical Co. Ltd. (Shanghai, China). Sodium selenite, Manganese (II) chloride tetrahydrate (MnCl_2_·4H_2_O), and anhydrous ethanol were purchased from the Sinopharm Chemical Reagent Co. Ltd. (Shanghai, China). The cell counting kit-8 (CCK-8) assay was obtained from Dojindo (Japan), and reactive oxygen species (ROS) assay kit was obtained from Nanjing Jiancheng Bioengineering Institute.

### Synthesis of BMMP

The biomass of pseudomonas geniculate cultures was evaluated according to the optical density at 600 nm (OD 600 nm). After reaching an optical density of 0.909 in 200 mL nutrient broth, the *Pseudomonas geniculate* was collected by centrifugation. Next, the obtained *Pseudomonas geniculate* was dispersed in 30 mL distilled water under magnetic stirring. Then, the suspension was treated with an ultrasonic cell disruptor for 30 min. Subsequently, the simple self-assembled biomimetic membrane obtained from *Pseudomonas geniculate* was collected and further rinsed with water, and named as BMMP.

### Synthesis of BMMP-Mn^2+^

After reaching an optical density of 0.909 in 200 mL nutrient broth, the *Pseudomonas geniculate* was collected by centrifugation. Next, the collected *Pseudomonas geniculate* and MnCl_2_·4H_2_O (49.48 mg) were dispersed in 30 mL distilled water under magnetic stirring for 10 h. Then, the above solution was treated with an ultrasonic cell disruptor for 30 min. Subsequently, the BMMP-Mn^2+^ was obtained after centrifugation, rinsing with water for three times, and vacuum freeze-drying.

### Synthesis of BMMP-Mn^2+^/Se

First, the Na_2_SeO_3_ (69.6 mg) was dissolved in 20 mL of distilled water under magnetic stirring for 30 min. Then, BMMP-Mn^2+^ (10 mg) was introduced and stirring was kept for 24 h. After which, the samples were collected and redispersed in 30 mL of water. Then, the mixed solution of Vc (10 mL, 70.4 mg) and chitosan (4 mL, 0.5 %) was dropwise added to the above resolution under magnetic conditions for 2 h. Finally, the BMMP-Mn^2+^/Se was collected and rinsing with water for three times.

### Synthesis of red cell membrane

First, the blood was obtained from mice, and the collected blood was transferred into a cuvette containing heparin sodium. Subsequently, the corresponding sediment in blood was collected and rinsing PBS, into which 0.2 mM EDTA was added. Next, PBS was again added to the solution to induce the occurrence of hemolysis. Finally, the red cell membranes (RCMs) were obtained by centrifugation.

### Loading and release of drug

BMMP-Mn^2+^/Se (10 mg) was dispersed in 10 mL DOX (2 mg/mL) solution under magnetic stirring for 24 h. Subsequently, the BMMP-Mn^2+^/Se loaded with DOX was obtained by centrifugation, and the loading mechanism of DOX was as follows: the surface of BMMP-Mn^2+^/Se exhibited the negative zeta potential, making it easily absorb DOX molecules with positive charge through electrostatic interaction, forming BMMP-Mn^2+^/Se/DOX nanoplatform. In addition, there are abundance of active groups on the surface of BMMP, which further enhanced the interaction between BMMP and DOX molecules through hydrogen bond. Then, the concentration of DOX released from BMMP-Mn^2+^/Se/DOX at different pH (pH = 7.4, 6.5, 5.5) was investigated. First, the BMMP-Mn^2+^/Se/DOX (10 mg) was dispersed in 10 mL of PBS at the three exact pH under shaking at a rate of 200 rpm. Then, 0.2 mL of supernatant was withdrawn at the predetermined time to measure the concentration of DOX released from the nanoplatform, and the same volume of solution was added to the tested solution.

### Hydrodynamic size change of nanoplatform in different media

BMMP-Mn^2+^/Se nanoparticles (3 mg) were dispersed in 5 mL of the distilled water, PBS, or FBS solution under ultrasonic treatment, respectively. Subsequently, the hydrodynamic size change of BMMP-Mn^2+^/Se was analyzed using dynamic light scattering (DLS) detector after different standing time. In addition, the measurement parameter of DLS condition was as follows: the index of refraction nanoparticles: 1.81, the refractive index: 1.33, high viscosity (20 °C): 1.002, and low viscosity (30 °C): 0.797.

### Confocal laser scanning observation

FITC was loaded into BMMP-Mn^2+^/Se/DOX according to the following methods. FITC (5 mg) was dissolved in water (10 mL) in dark environment for 30 min, into which was added with BMMP-Mn^2+^/Se/DOX (10 mg) under stirring for 24 h. Subsequently, FITC-labeled BMMP-Mn^2+^/Se/DOX was obtained after centrifugation and vacuum freeze-drying. In addition, HeLa cells were seeded on a confocal laser scanning microscope (CLSM)-specific dish (60 mm, 2 × 10^5^ cell/dish) and then incubated with the various concentrations of FITC-labeled BMMP-Mn^2+^/Se/DOX for 2, 4, and 8 h. Subsequently, 0.4% trypan blue was employed to quench extracellular fluorescence. Finally, fluorescence images of cells were obtained via CLSM (Zeiss LSM710 NLO, Germany), and the corresponding fluorescence intensity was calculated using FlowJo software (TreeStar).

### FITC accumulation assay

FITC-labeled BMMPs and FITC-labeled red cell membrane were employed to treat HeLa cells, respectively. Trypan blue at a concentration of 0.4% was used to quench extracellular fluorescence. Afterwards, the cells were analyzed by flow cytometry and the corresponding fluorescence intensity was calculated.

### MMP detection of HeLa cells

The mitochondrial membrane potential of HeLa cells was detected via the 5, 5′, 6, 6′-tetrachloro-1, 1′, 3, 3′-tetraethylbenzimidazolylcarbocyanine iodide (JC-1) assay. Briefly, HeLa cells were seeded into 96-well plates and further incubated with saline, Se, BMMP-Mn^2+^, and BMMP-Mn^2+^/Se (10 µg/mL) nanoparticles for 24 h. Subsequently, the HeLa cells were washed and then stained by JC-1 for 20 min. Finally, HeLa cells was observed by CLSM and flow cytometry. Meanwhile, the corresponding mitochondrial membrane potential was quantified via flow cytometry.

### Western blot analysis

First, HeLa cells were incubated in 6-well plates and then incubated with saline, Se, BMMP-Mn, BMMP/Se, and BMMP-Mn^2+^/Se (20 µg/mL) nanoparticles for 24 h. After the nanoparticle-treated HeLa cells were washed with PBS, the protein of cells was exacted. Subsequently, the amount of proteins in HeLa cells was detected via bicinchoninic acid protein quantitative kit. After the protein was separated by SDS-PAG, the corresponding protein was transferred to poly(vinylidene difluoride) membranes. Then, the membranes were respectively treated with SOD antibodies (dilution 1:500, 5 mL, 12 h), GPX4 antibodies (dilution 1:500, 5 mL, 12 h), cleaved caspase-3/9 (dilution 1:500, 5 mL, 12 h), and tubulin (dilution 1:2000, 5 mL, 12 h). Finally, the membranes were visualized via the chemiluminescence system.

### Cell culture and cytotoxicity assay

HeLa cells were seeded into 96-well plates (10^4^ cells/well) and incubated according to the standard protocols. Subsequently, HeLa cells were treated with BMMP, Se, DOX, BMMP-Mn^2+^, BMMP-Mn^2+^/Se, and BMMP-Mn^2+^/Se/DOX for 24 h. After PBS rinsing for three times, the cells were treated with 10 % CCK-8 kit (120 µL) at 37 °C for 2 h. Finally, the amount of live cells can be measured.

### Fenton-like catalytic activity and cellular ROS detection

The BMMP and BMMP-Mn^2+^/Se (1 mg/mL, 50 µL) were added to the mixed solution of 5,5-dimethyl-1-pyrroline N-oxide (DMPO, 50 mmol/L, 50 µL) and H_2_O_2_ (20 µL) at pH 5.5. Subsequently, the mixed solution was measured by electron spin resonance (ESR). In addition, the relative contents of ∙OH in solution were also measured through TMB method using UV–Vis spectrophotometer. First, BMMP-Mn^2+^/Se were added to the mixed PBS solution (pH 5.5) containing H_2_O_2_ (60 µL) and TMB (200 µL) at different concentrations (0, 40, 80, 160, 320, 640, 1280 µg/mL). After 3 min, the nanoparticles were centrifuged, and the supernatant was collected and then measured using UV–Vis spectrophotometer. Similarly, the pH-dependent catalytic process were carried as follows: BMM-Mn^2+^/Se (1 mg/mL, 50 µL) was uniformly dispersed into the medium (3 mL) containing H_2_O_2_ (60 µL, 28 %) and TMB (200 µL) at different pH conditions. Afterwards, the nanoparticles were centrifuged and the supernatant was collected and then measured using UV–Vis spectrophotometer.

In addition, HeLa cells were seeded on glass coverslips and further treated with physiological saline (control group), BMMP, Mn^2+^, Se, BMMP-Mn^2+^, and BMMP-Mn^2+^/Se for 4 h. Then, DCFH-DA prods (dilution 1:1500) were employed to treat HeLa cells at 37 ^o^C for 30 min. The corresponding cells were washed and further fixed in paraformaldehyde (4 %) solution. Subsequently, HeLa cells were treated with DAPI (dilution 1:2000) fluorescent dye for 10 min. Finally, the intracellular ROS was observed by CLSM and the corresponding contents of ROS in cells were quantified via flow cytometry.

### Biodistribution of nanoparticles in vivo

Xenografted cervical tumor-bearing mice were treated with BMMP-Mn^2+^/Se/DOX (10 mg/kg). Afterward, the mice were sacrificed at different time, and the corresponding tissues were collected and then treated with a certain concentrations of nitric acid for 72 h. Finally, the Se concentrations were quantified.

### Anticancer activity assay

Mice used in this study were treated in accordance with the ethics committee guidelines at the University of Science and Technology of China. The cervical tumor-bearing mice were divided into six groups (n = 5/group) and these mice were respectively injected with saline, Se, DOX, BMMP, BMMP-Mn^2+^, BMMP-Mn^2+^/Se, BMMP-Mn^2+^/DOX, and BMMP-Mn^2+^/Se/DOX (2 mg/kg) through the tail vein at 2-day intervals. The volume of tumor (V) was calculated by the formula: $\text{V}=\text{a} \times {\text{b}^2}/2,$ where “a” and “b” are the largest and smallest tumor dimension, respectively, and the mice were weighted.

### Pathological analysis

The tissues obtained from the mice treated with nanoparticles were fixed in 10% buffered formalin for 24 h, and then embedded in paraffin. Subsequently, the corresponding tissues were cut into two halves. One half was treated with H&E and was observed by an inverted florescence microscope system. The other half was subject to immunohistochemical detection of cleaved caspase-3, and the corresponding specific experimental steps were similar to those in previously reported studies.

### Pharmacokinetic experiment

The mice (n = 3) were injected with nanoparticles (BMMP-Mn^2+^/Se/DOX) at a dosage of 10 mg/kg via tail vein. Subsequently, the blood, urine, and feces of mice were collected at different interval time, and then were nitrated to form transparent solution using the concentrated nitric acid. After that, the resulting solution was diluted, and then filtered through 220 nm of filter membrane. Finally, Se content in the solution was analyzed via inductively coupled plasma optical emission spectrometer (ICP-OES).

## Results and discussion

**Fig. 1:**
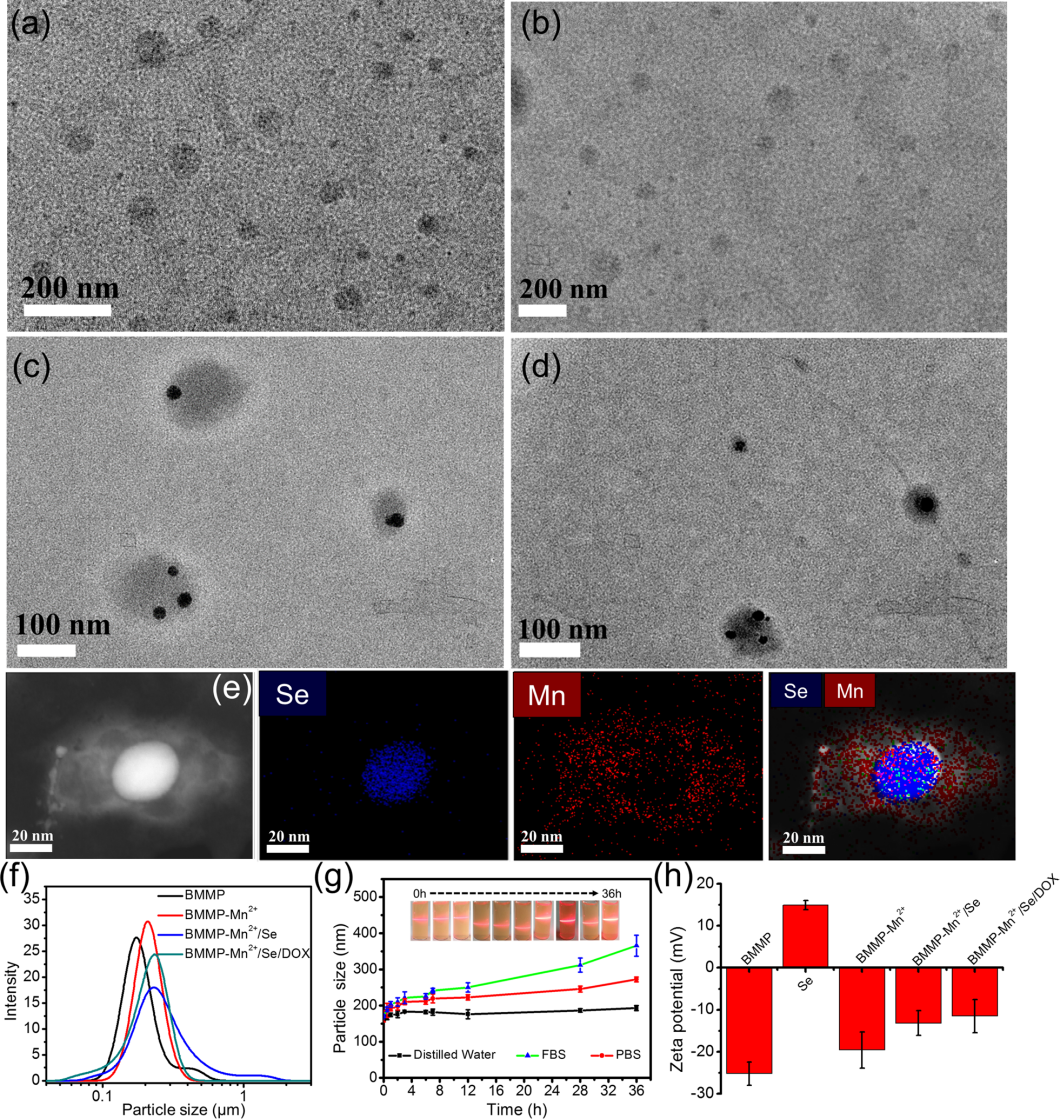
TEM images of **a** BMMP, **b** BMMP-Mn^2+^, **c** BMMP-Mn^2+^/Se, **d** BMMP-Mn^2+^/Se/DOX and **e** the elemental area mapping of BMMP-Mn^2+^/Se. **f** The hydrodynamic size distribution of nanoparticles in distill water. **g** The average sizes of BMMP-Mn^2+^/Se with standing time increasing in distilled water, FBS, and PBS solution. **h** The zeta potential of nanoparticles

BMMP and BMMP-Mn^2+^ could be obtained through ultrasonic treatment for the mixture of *Pseudomonas geniculate* without or with the Mn compound. The morphologies of BMMPs and BMMP-Mn^2+^ showed significant microvesicles with sizes of approximately 90 and 95 nm (Fig. 1a, b), respectively. In addition, Small Se nanoparticles (approximately 20 nm) were synthesized using a classical reduction of sodium selenite, and could be successfully loaded into BMMP-Mn^2+^ microvesicles (Fig. 1c). Hydrodynamic size results indicated that BMMP-Mn^2+^/Se was larger than BMMP-Mn^2+^, which might be attributed to the aggregation and vesicle fusion of nanoparticles (Fig. 1f). As shown in Fig. 1g, the hydrodynamic size of BMMP-Mn^2+^/Se in distilled water hardly changed with standing time increasing. When in the fetal bovine serum (FBS) and PBS solution, the size of BMMP-Mn^2+^/Se slightly increased with increasing standing time, but the solution of BMMP-Mn^2+^/Se still had a significant Tyndall effect. Therefore, the above results demonstrated that BMMP-Mn^2+^/Se nanoplatform had excellent stability in these media. To further confirm the synthesis process, the zeta potential of the nanoplatform was measured as shown in Fig. 1h. Se nanoparticles showed a significant positive charge due to the cationic polymer coating. The zeta potential of BMMP-Mn^2+^/Se significantly decreased compared to that of pure BMMP, suggesting that Se nanoparticles were successfully loaded into BMMP-Mn^2+^ through electrostatic interactions. Subsequently, the energy-dispersive X-ray (EDX) spectrum of BMMP-Mn^2+^/Se also confirmed the presence of Mn and Se elements (Additional file 1: Fig. S1). Similarly, element mapping analysis found that Mn species were homogeneously distributed into BMMP-Mn^2+^/Se, and Se nanoparticles mainly existed in the inner zone (Fig. 1e).

**Fig. 2:**
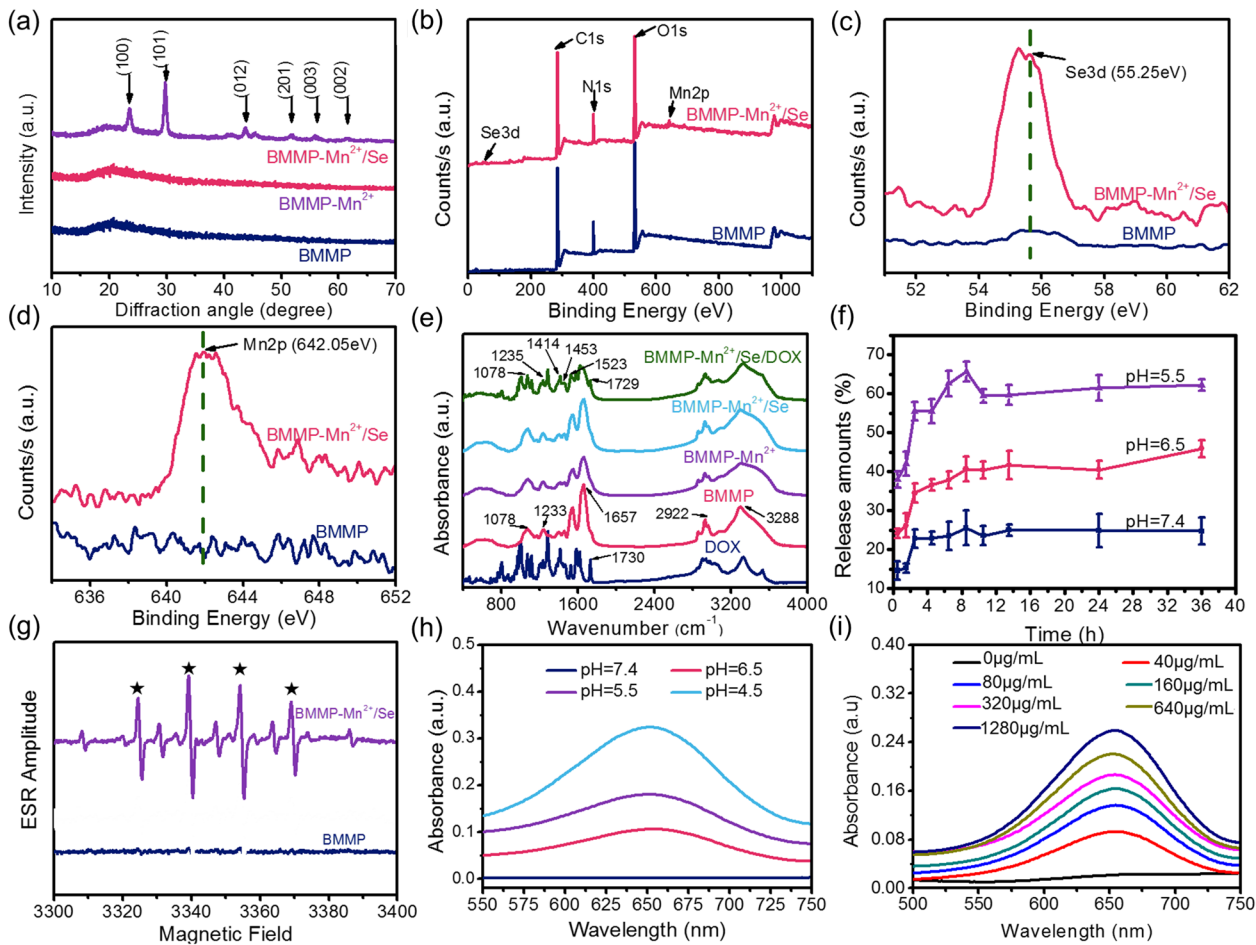
**a** XRD of BMMP, BMMP-Mn^2+^, and BMMP-Mn^2+^/Se. **b** XPS full spectra, **c** Se 3d spectra, and **d** Mn 2p spectra of BMMP and BMMP-Mn^2+^/Se. **e** FT-IR spectra of nanoparticles. **f** The release behavior of DOX from BMMP-Mn^2+^/Se/DOX under different pH solution. **g** ESR spectra of the mixed solution (nanoparticles, DMPO, H_2_O_2_). **h** The UV absorption spectra of mixed solutions (nanoparticles, TMB, H_2_O_2_) with different pH values, and **i** the BMMP-Mn^2+^/Se mixed with TMB and H_2_O_2_ in pH 5.5 solution

The X-ray diffraction (XRD) pattern indicated that BMMP-Mn^2+^ and BMMP had no significant peaks as shown in Fig. 2a, suggesting that the structures of BMMP-Mn^2+^ and BMMP were amorphous. However, significant diffraction peaks could be observed for BMMP-Mn^2+^/Se (JCPDS no. 73-0465), implying that the Se nanoparticles were successfully equipped with the BMMP-Mn^2+^ nanoplatform. In addition, the valence states of Mn, Se, C, N, and O elements in BMMP-Mn^2+^/Se was verified by X-ray photoelectron spectroscopy (XPS) (Fig. 2b). The Se3d peak that appeared at 55.25 eV was assigned to the zero valence of Se [45], which confirmed the existence of selenium (Fig. 2c). In addition, the Mn2p peak at 642.05 eV was assigned to the Mn^2+^ signal [46], implying that the BMMP-Mn^2+^/Se nanoplatform had the potential to catalyze Fenton-like reaction (Fig. 2d). Subsequently, the loading amounts of Mn and Se in nanoplatform were measured to be 6.27 and 3.2%, respectively. DOX, a classical anticancer drug, was further loaded into BMMP-Mn^2+^/Se to fabricate a chemotherapeutic- and chemodynamic therapeutic nanoplatform. After loading with DOX in the BMMP-Mn^2+^/Se nanoplatform, new peaks at 1523, 1453, and 1414 cm^− 1^ could be ascribed to the skeleton vibration of benzene ring in DOX compared to other samples (Fig. 2e), implying that DOX was loaded into BMMP-Mn^2+^/Se. In addition, the morphology and hydrodynamic size of BMMP-Mn^2+^/Se/DOX were similar to those of BMMP-Mn^2+^/Se nanoparticles (Fig. 1d, f). Subsequently, the loading efficiency of DOX was investigated by ultraviolet visible (UV–Vis) analysis. The absorption peak at 496 nm characteristic of DOX, in the supernatant significantly decreased after BMMP-Mn^2+^/Se treatment, implying that DOX was effectively loaded. The loading capacity of DOX was also calculated and was approximately 260.64 µg/mg (Additional file 1: Fig. S2). Notably, whether DOX could be released from BMMP-Mn^2+^/Se/DOX in the tumor microenvironment was an important indicator. As shown in Fig. 2f, the release amount of DOX increased significantly with the pH value of the solution decreasing and showed a pH-dependent relationship. BMMP-Mn^2+^/Se/DOX showed a low release amount at pH 7.4, which could effectively decrease the side effects of DOX on normal tissue.

It is known that Mn^2+^ ion can catalyze H_2_O_2_ to hydroxyl radicals (·OH) via a Fenton-like reaction [47]. Due to the paramagnetism of ·OH, ·OH concentrations were detected through electron spin resonance (ESR). First, 5, 5-dimethyl-1-pyrroline N-oxide (DMPO) was employed to capture ·OH and then form a stable ·OH/DMPO. As shown in Fig. 2g, a hyperfine splitting constant assigned to the characteristic spectra of ·OH/DMPO appeared after BMMP-Mn^2+^/Se treatment, indicating that BMMP-Mn^2+^/Se could be an excellent chemodynamic therapy agent (CDTA) for effectively catalyzing H_2_O_2_ to ·OH. Simultaneously, 3,3′,5,5′-tetramethylbenzidine (TMB) was also employed to observe ·OH generation. The color of solution gradually turned from colorless to blue as the pH value of solution decreased and the concentration of BMMP-Mn^2+^/Se increased, and the corresponding absorption intensity also gradually increased, suggesting that the catalytic ability of BMMP-Mn^2+^/Se was concentration- and pH-dependent (Additional file 1: Fig. S3a, b and Fig. 2h, i). Based on the above analysis, we considered that BMMP-Mn^2+^/Se could play an excellent role in catalyzing the Fenton-like reaction and then accelerate cancer cell death.

**Fig. 3:**
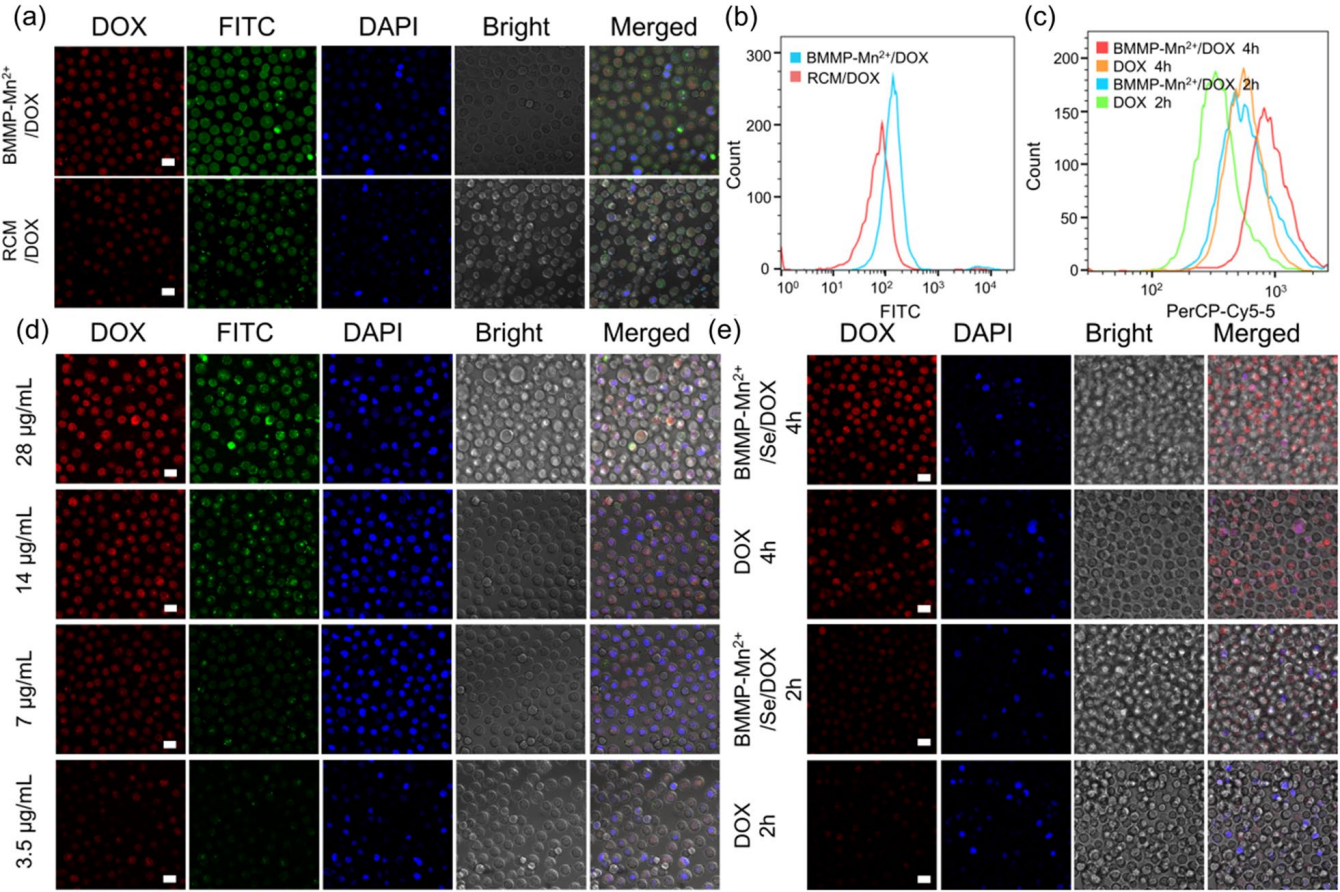
**a** The CLSM images of HeLa cells treated with FITC-labeled RCM/DOX and BMMP-Mn^2+^/DOX at same concentration, and **b** the corresponding flow cytometry analysis, the same scale bar applies to all images. Scale bar: 20 μm. **c** The flow cytometry analysis of HeLa cells treated with free DOX and BMMP-Mn^2+^/Se/DOX at same concentration (DOX: 1 µg/mL). **d** The CLSM images of HeLa cells treated with FITC-labeled BMMP-Mn^2+^/Se/DOX for 4 h, the same scale bar applies to all images. Scale bar: 20 μm. **e** The CLSM images of HeLa cells treated with free DOX and BMMP-Mn^2+^/Se/DOX at same concentration (DOX: 1 µg/mL)

The cellular uptake of nanoparticles was observed via confocal laser scanning microscopy (CLSM). First, we compared the cellular uptake of red cell membranes (RCMs) and BMMP-Mn^2+^. As shown in Fig. 3a, HeLa cells treated with FITC-labeled BMMP-Mn^2+^/DOX exhibited stronger red fluorescence and green fluorescence than that with FITC-labeled RCM/DOX. In addition, the corresponding flow cytometry analysis data also showed the same results with CLSM observation (Fig. 3b). These results demonstrated that BMMP-Mn^2+^ nanoplatform could be more effectively internalized by cancer cells than traditional membrane-based DDSs. The internalization efficiency is greater; thus, more drugs would be delivered to cancer cells. Subsequently, the accumulation of DOX in cells was also analyzed through CLSM observation. HeLa cells in DOX group showed slight red fluorescence, and the fluorescence intensity was positively correlated with the incubation time (Fig. 3c, e). Nevertheless, the accumulation of DOX in cells significantly increased after BMMP-Mn^2+^/Se/DOX treatment compared to free DOX. This result demonstrated that BMMP-Mn^2+^ with efficient internalization could deliver more DOX molecules into cancer cells. Therefore, BMMP-Mn^2+^/Se/DOX could be a promising candidate as an excellent cancer therapeutic nanoplatform.

Subsequently, to further verify the effective internalization of BMMP-Mn^2+^/Se/DOX, BMMP-Mn^2+^/Se/DOX was rationally labeled by FITC, with which HeLa cells were incubated. As shown in Fig. 3d, intracellular green and red fluorescence gradually increased as the concentration of BMMP-Mn^2+^/Se/DOX increased. Moreover, red fluorescence and green fluorescence showed colocalization in cells, suggesting that HeLa cells could quickly ingested BMMP nanoplatform. In addition, the uptake of nanoplatform was quantitatively analyzed by flow cytometry and showed a dose-dependent relationship (Additional file 1: Fig. S4a). Subsequently, we further observed cell uptake at different incubation times, and the corresponding results indicated that the fluorescence intensity in cells increased with increasing incubation time (Additional file 1: Fig. S4b, d). These results demonstrated that BMMP-Mn^2+^/Se/DOX could effectively deliver more drugs to cancer cells, which would be beneficial to achieve better anticancer effects. In order to explore the pathway of internalization, the cell uptake of BMMP-Mn^2+^/Se/DOX was analyzed through flow cytometry at low temperature and endocytosis inhibitors (chlorpromazine and amiloride) treatment (Additional file 1: Fig. S4c). It could be seen that the cell uptake of BMMP-Mn^2+^/Se/DOX did not significantly decrease after amiloride treatment, indicating that the pinocytosis was not a main endocytic pathway. Notably, the uptake of BMMP-Mn^2+^/Se/DOX dramatically decreased after 4 °C and chlorpromazine treatment, indicating that the pathway of BMMP-Mn^2+^/Se/DOX entered cells might be energy- and clathrin-mediated endocytosis.

**Fig. 4:**
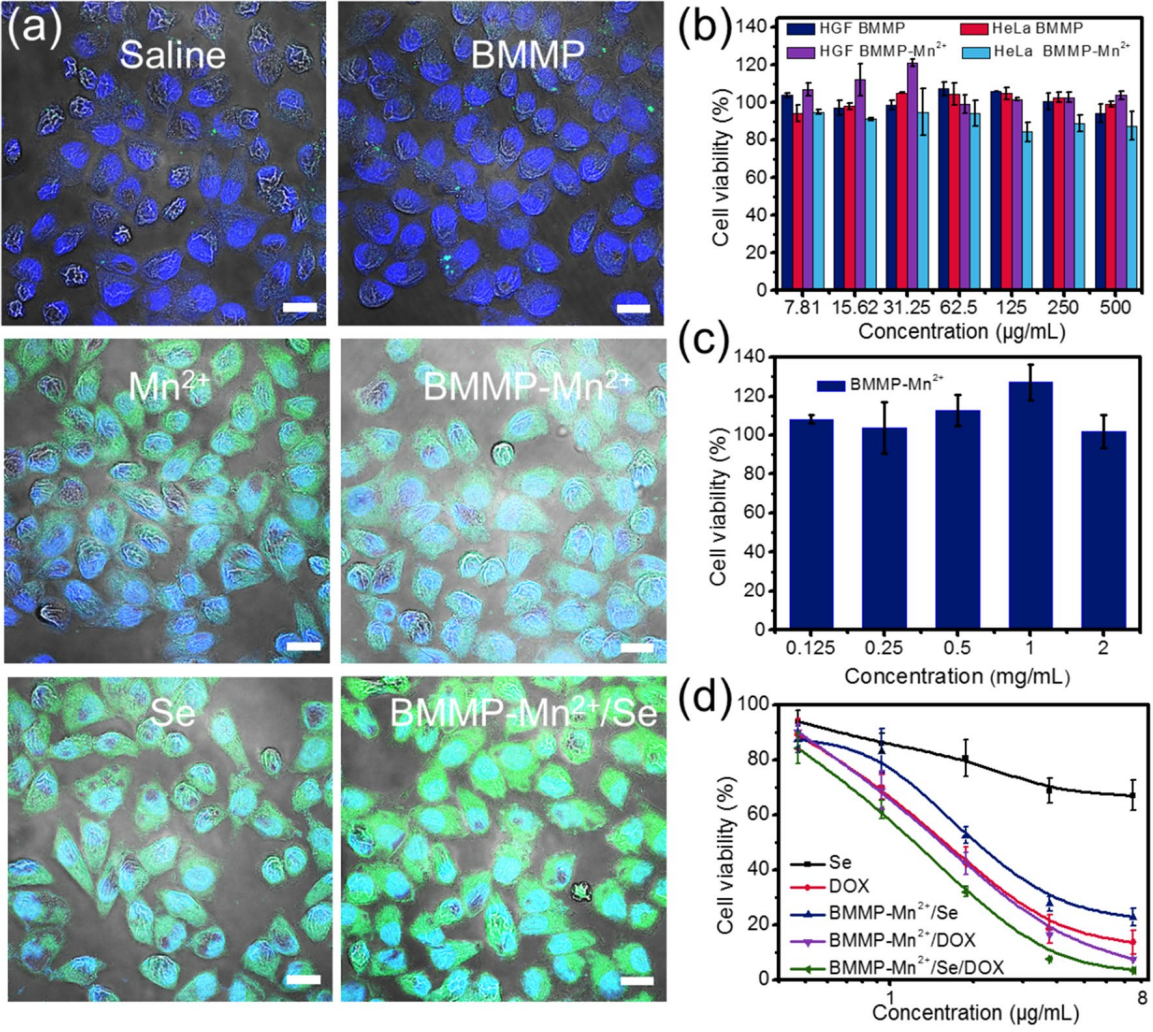
**a** CLSM observation of intracellular ROS in HeLa cells, the same scale bar applies to all images. Scale bar: 20 μm. **b** The viability of HGF and HeLa cells incubated with BMMPs and BMMP-Mn^2+^ for 24 h and **c** the viability of HGF cells incubated with BMMP-Mn^2+^ for 24 h. **d** The viability of HeLa cells treated with different nanoparticles for 24 h

The intracellular ROS generation was investigated through the detection of 2′,7′-dichlorodihydrofluorescein diacetate (DCFH-DA) after BMMP-Mn^2+^/Se treatment. In Fig. 4a, saline- and BMMP-treated HeLa cells showed negligible green fluorescence, but Mn^2+^ ion- and Se-treated HeLa cells exhibited significant green fluorescence, implying that both Mn^2+^ ions and Se could activate intracellular ROS generation. In particular, HeLa cells in BMMP-Mn^2+^/Se group exhibited the brightest green fluorescence among all other groups, indicating that Mn^2+^ and Se in BMMP-Mn^2+^/Se possessed significant synergistic action to enhance intracellular ROS generation. In addition, ROS levels in HeLa cells were quantitatively analyzed via flow cytometry. The fluorescence intensity of HeLa cells showed the following order: BMMP-Mn^2+^/Se > BMMP-Mn^2+^ > Se > free Mn^2+^ > BMMP > saline. This findings further suggest that BMMP -Mn^2+^/Se exhibited the strongest ability to activate intracellular ROS generation and enhance anticancer activity (Additional file 1: Fig. S5).

In addition, the biocompatibility of nanoplatform was further investigated using a classical CCK-8 assay. The viability of BMMP- or BMMP-Mn^2+^-treated HGF cells had no significant decrease, indicating that both BMMP and BMMP-Mn^2+^ exhibited excellent biocompatibility (Fig. 4b). Moreover, BMMP-Mn^2+^ also caused no significant toxicity to HGF cells at a wide range of concentrations as shown in Fig. 4c. This feature is beneficial to assess the mechanism of action for BMMP-Mn^2+^/Se/DOX. In addition, HGF cell morphology did no exhibited significant variation after BMMP-Mn^2+^ treatment, further confirming its excellent biocompatibility (Additional file 1: Fig. S6). When the nanoplatform entered cells, the inhibitory effect of BMMP-Mn^2+^/Se/DOX was investigated (Fig. 4d). A dose-dependent effect on cell viability was noted in all groups of treated cells. Both Se nanoparticles at low concentrations slightly decreased cell viability, but BMMP-Mn^2+^/Se could significantly promote the cancer cell apoptosis at low concentration, and achieving an approximately 80% apoptosis rate. This was ascribed to the effective synergistic anticancer effect of Mn^2+^ ions and Se nanoparticles. When loaded with DOX, the inhibitory effect of BMMP-Mn^2+^/Se on cancer cells was strengthened, suggesting that the combined therapy possessed a better anticancer ability. Overall, the ability to induce cell death exhibited the following order: BMMP-Mn^2+^/Se/DOX > BMMP-Mn^2+^/DOX > DOX > BMMP-Mn^2+^/Se > Se. The IC_50_ value of BMMP-Mn^2+^/Se/DOX was lowest compared to other samples (Additional file 1: Table S1). These results demonstrated that DOX-loaded BMMP-Mn^2+^/Se possessed the strongest therapy compared to other nanoparticles. As previously reported [44, 48, 49], ROS-induced cell death was different from chemotherapy-induced death because it could escape the biological barrier and overcome cancer resistance. Based on this finding, we further investigated the ability of BMMP-Mn^2+^/Se/DOX to overcome cancer resistance (Additional file 1: Fig. S7). Both BMMP-Mn^2+^/Se/DOX and free DOX showed strong inhibition on MCF-7 cells viability. However, for MCF-7/ADR cells, the inhibition ability of free DOX was significantly decreased, which was attributed to the multidrug resistance of MCF-7/ADR cells. It was noted that BMMP-Mn^2+^/Se/DOX showed a similar inhibition ability in MCF-7 and MCF-7/ADR cells, suggesting that BMMP-Mn^2+^/Se/DOX could effectively overcome the drug resistance of cancer cells.

**Fig. 5:**
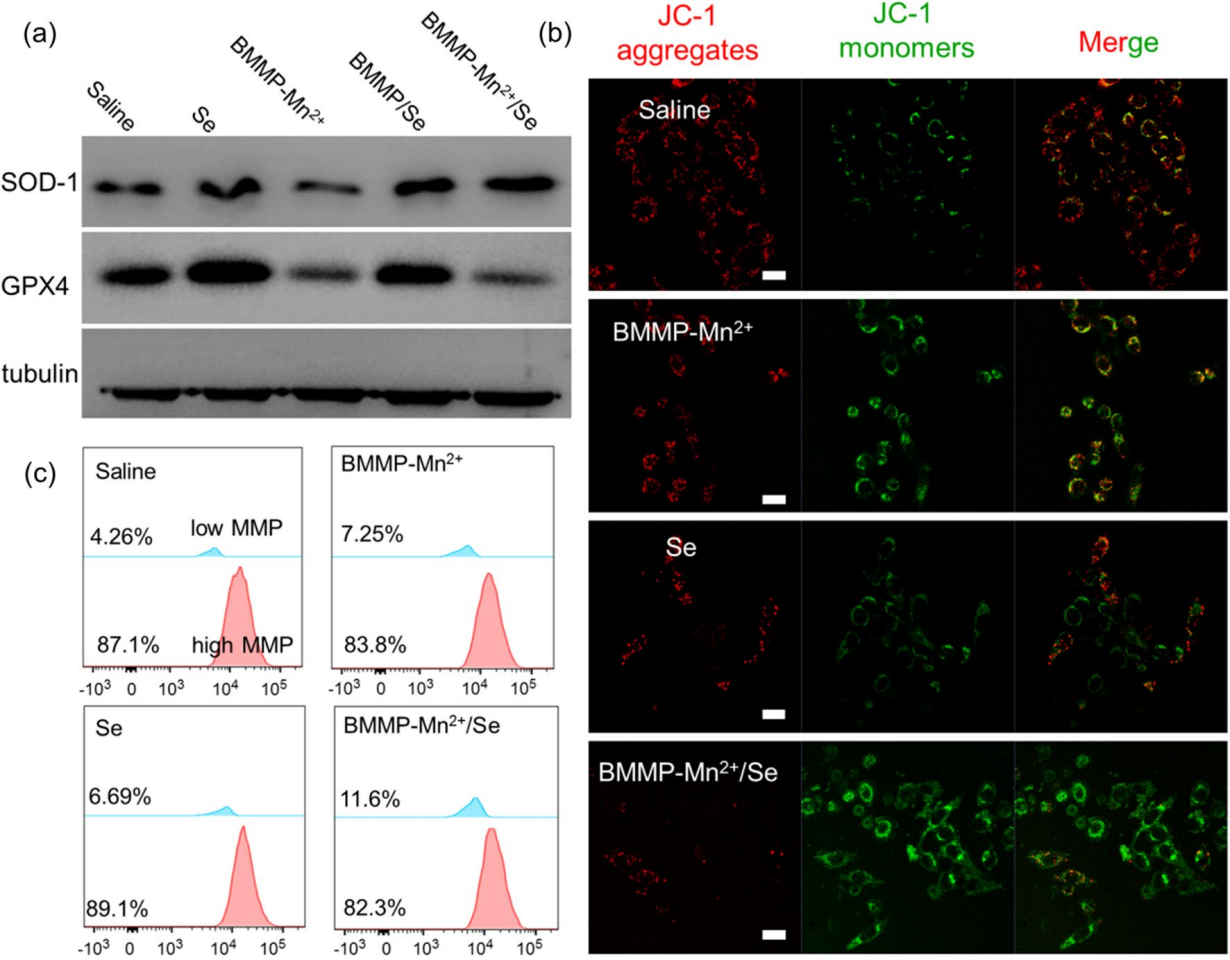
**a** The protein expression of HeLa cells incubated with different samples. **b** Detection of MMP by fluorescence microscopy in HeLa cells using the JC-1 probe, the same scale bar applies to all images. Scale bar: 20 μm. **c** Qualitative analysis of MMP in HeLa cells

We considered that the effective synergistic effect of BMMP-Mn^2+^/Se mainly originated from SOD-1 activation and GPX4 inactivation. Based on this analysis, SOD-1 and GPX4 expression was investigated through western blot analysis as shown in Fig. 5a. After Se, BMMP-Mn^2+^, BMMP/Se, and BMMP-Mn^2+^/Se treatment, the expression of SOD-1 in BMMP-Mn^2+^-treated cells had no significant variation, but these nanopatform contained Se could dramatically promote SOD-1 expression, which could further promote the elimination of intracellular O_2_^**−·**^ and downstream H_2_O_2_ generation (Additional file 1: Fig. S8). Subsequently, the generated H_2_O_2_ could be effectively catalyzed by the Fenton-like reaction and subsequently formed highly toxic ·OH. Notably, compared to the saline group, the GPX4 expression of HeLa cells significantly decreased after BMMP-Mn^2+^ and BMMP-Mn^2+^/Se treatment. Due to the reduction in GPX4 expression, ROS could not be eliminated from cancer cells in a timely manner, which dramatically enhanced intracellular ROS accumulation. Meanwhile, the results of quantitative analysis also confirmed that BMMP-Mn^2+^/Se could activate the expression of SOD-1 and simultaneously inhibit the expression of GPX4 in HeLa cells (Additional file 1: Fig. S9), and it could further increase the oxidative stress at the cellular level. Enhanced oxidative stress could significantly cause mitochondrial damage and further induce the apoptotic cascade. The mitochondrial membrane potential (MMP) was measured using JC-1 dye to observe any damage (Fig. 5b, c). For normal cells with high MMP, intracellular JC-1 dye spontaneously formed JC-1 aggregates to emit red fluorescence. Nevertheless, when JC-1 dye entered damaged cells, MMP significantly decreased, causing JC-1 dye to remain monomeric and emit green fluorescence. The mitochondria of HeLa cells treated with saline exhibited strong red fluorescence and low green fluorescence, and the corresponding low MMP was only 4.26%, indicating that HeLa cells possessed excellent viability. In addition, BMMP-Mn^2+^ and Se-treated cells showed relatively strong green fluorescence, and the low MMP also increased to 7.25 and 6.69%, respectively. This result indicated that BMMP-Mn^2+^ and Se nanoparticles could partly cause mitochondrial damage. Notably, BMMP-Mn^2+^/Se-treated cells exhibited the strongest green fluorescence and the lowest MMP, implying that BMMP-Mn^2+^/Se possessed the strongest mitochondrial damage effect. These results were consistent with ROS generation, demonstrating that mitochondrial damage was mainly triggered by the ROS pathway. Mitochondrial damage could activate the expression of apoptotic enzymes (i.e., cleaved caspase-3/9), inducing cancer cell death. Subsequently, the expression level of cleaved caspase-3/9 was analyzed. BMMP-Mn^2+^/Se-treated HeLa cells exhibited the highest expression of cleaved caspase-3/9 compared to other groups (Additional file 1: Fig. S10), suggesting that BMMP-Mn^2+^/Se could effectively induce cancer cell death via the mitochondria-mediated apoptotic pathway.

**Fig. 6:**
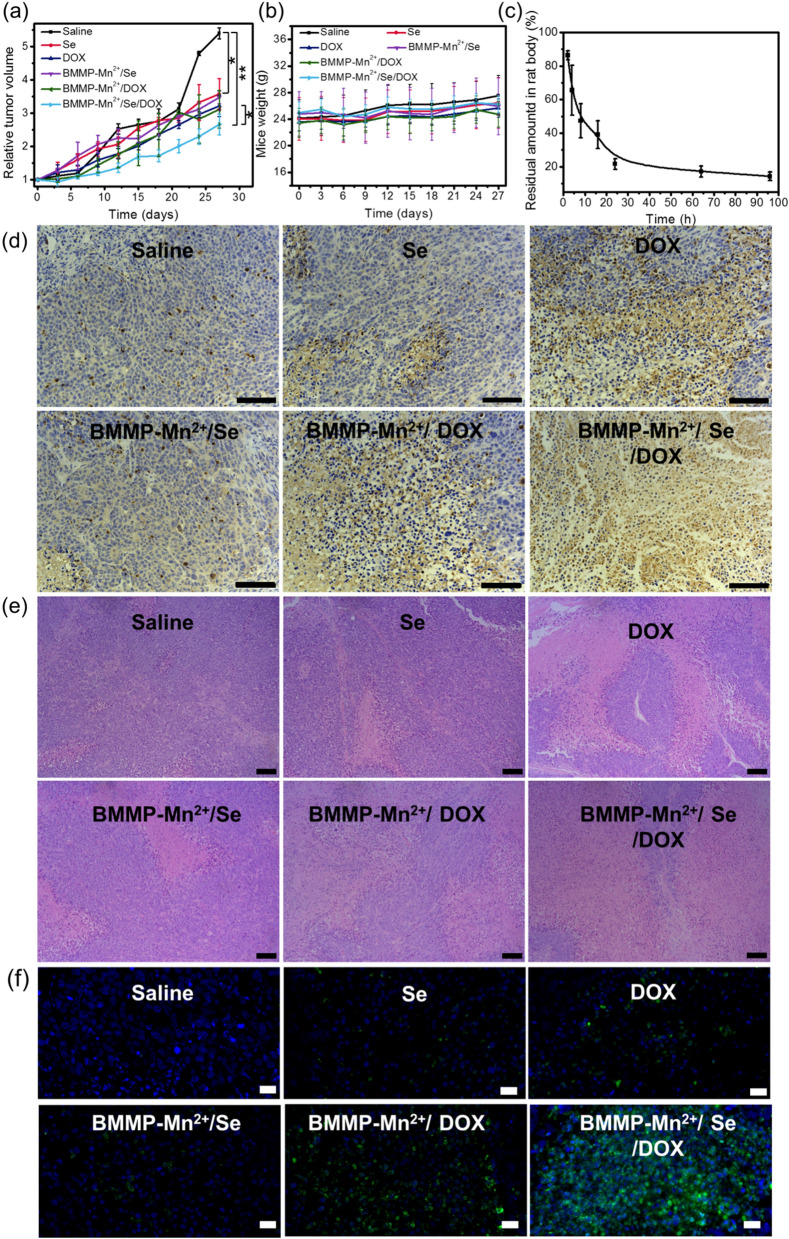
**a** The relative tumor volume and **b** the corresponding body weight of mice after injecting different nanoparticles. **c** Blood elimination kinetics of BMMP-Mn^2+^/Se/DOX in vivo. **d** Immunohistochemical staining images and **e** H&E staining of tumor tissues. Scale bar: 100 μm. **f** The TUNEL assay of tumor tissues. Scale bar: 20 μm. *Denotes 0.01 < p < 0.05 and ** denotes p < 0.01

To assess the application potential of the therapeutic nanoplatform, DOX-loaded BMMP-Mn^2+^/Se was intravenously injected into tumor-bearing BALB/C nude mice (2 mg/kg). In addition, in order to illustrate the anticancer ability of BMMP-Mn^2+^/Se/DOX, some control groups including saline, Se, DOX, BMMP-Mn^2+^/Se, and BMMP-Mn^2+^/DOX were also used to treat cancer-bearing mice through tail vein injection. The relative tumor volumes of mice were recorded after each treatment at 2-day intervals. As shown in Fig. 6a, the relative tumor volume in mice treated with saline significantly increased with increasing time, and reached approximately 5.4. Compared to the saline group, Se, DOX, BMMP-Mn^2+^/Se, BMMP-Mn^2+^/DOX, and BMMP-Mn^2+^/Se/DOX groups showed different degrees of inhibitory effects on tumor growth. Notably, the tumor growth in BMMP-Mn^2+^/Se/DOX group was significantly inhibited and its relative volume was less than 2.7, indicating that BMMP-Mn^2+^/Se/DOX exhibited the strongest anticancer ability compared to the other groups. This finding also demonstrated that the combination of CDT and chemotherapy could synergistically promote cancer cell death and provide a better anticancer pathway [50, 51]. The photographs of tumor-bearing mice and solid tumors (Additional file 1: Fig. S11) revealed that the tumor sizes of mice treated with BMMP-Mn^2+^/Se/DOX were the smallest, further confirming that the BMMP-Mn^2+^/Se/DOX had the strongest tumor inhibition ability.

Subsequently, the expression level of cleaved caspase-3 in tumor sections was analyzed through immunohistochemical staining analysis (Fig. 6d). The tumor slices treated with saline minimally expressed cleaved caspase-3. In addition, Se and free DOX only showed low expression of cleaved caspase-3 in tumor slices, but BMMP-Mn^2+^/Se and BMMP-Mn^2+^/DOX significantly exhibited higher expression of cleaved caspase-3, indicating that the BMMP-Mn^2+^ carrier could deliver more drug into tumor tissue and subsequently induce more tumor cell death. Notably, the tumor slices treated with BMMP-Mn^2+^/Se/DOX showed the highest expression of cleaved caspase-3, further confirming that the integration of CDT and chemotherapy could induce more apoptosis in tumor tissue. Meanwhile, the corresponding quantitative analysis also confirmed the above result (Additional file 1: Fig. S12). H&E staining analysis showed the following necrotic region pattern (Fig. 6e): BMMP-Mn^2+^/Se/DOX > BMMP-Mn^2+^/DOX > DOX > BMMP-Mn^2+^/Se > Se > saline. In addition, the density of hyperchromatin tumor cells injected with BMMP-Mn^2+^/Se/DOX was less than that of other groups, but the quantity of atypical nuclei in BMMP-Mn^2+^/Se/DOX group was greater compared to other groups. The apoptosis/necrosis regions in tumor tissues were also investigated via the TUNEL assay (Fig. 6f). Massive TUNEL-positive cells (green fluorescence) were widely distributed in tumor tissues treated with BMMP-Mn^2+^/Se/DOX, BMMP-Mn^2+^/DOX, DOX, BMMP-Mn^2+^/Se, and Se nanoparticles, implying that these nanoparticles could induce the different degrees of cell apoptosis in tumors. Notably, the BMMP-Mn^2+^/Se/DOX-treated tumor tissues in the group exhibited the brightest green fluorescence, demonstrating that BMMP-Mn^2+^/Se/DOX nanoparticles possessed the best inhibition ability for tumors compared to other nanoparticles, which was consistent with the H&E results.

Subsequently, the weight of tumor-bearing mice was recorded as shown in Fig. 6b. The body weights of mice treated with different samples were not significantly different, implying that BMMP-Mn^2+^/Se/DOX could not cause systemic toxicity and showed excellent biosafety. Subsequently, the biodistribution analysis demonstrated that BMMP-Mn^2+^/Se/DOX mainly accumulated in the spleen, kidney, and liver, which might be ascribed to the existence of the reticuloendothelial system (RES) in these organ tissues. Notably, BMMP-Mn^2+^/Se/DOX could quickly accumulate in tumor tissue based on the enhanced permeability and retention effect (EPR) at 8 h postinjection (Additional file 1: Fig. S13), which was beneficial to promote cancer therapy with the nanoplatform. Subsequently, the pharmacokinetics of BMMP-Mn^2+^/Se/DOX were also investigated by measuring Se levels. As shown in Fig. 6c, approximately 50% of BMMP-Mn^2+^/Se/DOX was retained in the body at 8 h postinjection, implying that BMMP-Mn^2+^/Se/DOX had a long blood circulation time. Meanwhile, BMMP-Mn^2+^/Se/DOX was also completely excreted from the body at 96 h postinjection. These results indicated that BMMP-Mn^2+^/Se/DOX had long-acting effects on tumors and that its residue did not cause obvious side effects. To verify the biosafety of nanoplatform, H&E staining of critical organs was conducted after treatment withdrawal. The heart slices of mice treated with free DOX exhibited some abnormalities (Additional file 1: Fig. S14), such as the pyknotic nuclei in myocardial cells. In addition, these critical organs in other groups showed normal histomorphology and had no obvious damage. These results demonstrated that the BMMP-Mn^2+^/Se/DOX nanoplatform exhibited excellent biosafety.

## Conclusions

In summary, a self-assembled biomimetic cytomembrane-based nanoplatform for chemotherapeutic- and chemodynamic synergistic therapy of cancer based on loading with Se and DOX was developed in this study. This nanoplatform (BMMP-Mn^2+^/Se/DOX) could be easily prepared via a facile and simple ultrasonic treatment. In addition, compared to traditional RCM, this nanoplatform showed more effective internalization efficiency of cancer cells to promote anticancer drug delivery and subsequently enhance the therapeutic effect. Moreover, this nanoplatform could activate SOD-1 expression, and then upregulate downstream H_2_O_2_ levels. In addition, Mn^2+^ in the nanoplatform could further catalyze H_2_O_2_ to highly toxic ·OH in vivo. Interestingly, the BMMP-Mn^2+^/Se nanoplatform inhibits GPX4 expression, accelerating ROS accumulation in cells. The accumulated ROS induced mitochondrial damage and subsequently induced cancer cells apoptosis, achieving effective CDT for cancer. In addition, the loading of the classical anticancer drug DOX in the nanoplatform could endow the BMMP-Mn^2+^/Se/DOX nanoplatform with chemotherapeutic effects, which could further promote the therapeutic efficacy of this platform in the treatment of cancer. Through in vivo experiments, the systemic delivery of nanoplatforms exhibited low toxicity in normal organs, but had a strong inhibitory effect on tumor growth. Therefore, this nanoplatform provides a promising candidate to fabricate chemotherapeutic- and chemodynamic synergistic therapy systems using membrane-based nanocarriers.

## Supplementary Information

**Additional file 1: Fig. S1.** The EDX spectrum of BMMP-Mn^2+^/Se. **Fig. S2.** The UV absorption spectra of DOX before and after loading. **Fig. S3.** (a) The color changes of the mixture of BMMP-Mn^2+^/Se in different pH solutions and (b) the different concentration of BMMP-Mn^2+^/Se at pH 5.5 solution. **Fig. S3.** The UV absorption spectra of different nanoparticles mixed with TMB, and H_2_O_2_ in solution. **Fig. S4.** (a) The flow cytometry analysis of HeLa cells treated with FITC-labeled BMMP-Mn^2+^/Se/DOX at different concentration. (b) The flow cytometry analysis HeLa cells treated with FITC-labeled BMMP-Mn^2+^/Se/DOX for different time. (c) The flow cytometry analysis for endocytosis pathway of BMMP-Mn^2+^/Se/DOX nanoparticles. (d) The CLSM images of HeLa cells treated with FITC-labeled BMMP-Mn^2+^/Se/DOX for different time, the same scale bar applies to all images. Scale bar: 20 μm. **Fig. S5.** The flow cytometry analysis of intracellular ROS in HeLa cells. **Fig. S6.** The morphology of HGF cells treated with different concentrations of BMMP-Mn^2+^ for 24 h. Scale bar: 100 μm. **Fig. S7.** The viability of (a) MCF-7 and (b) MCF-7/ADR cells treated with free DOX and BMMP-Mn^2+^/Se/DOX for 24 h. **Fig. S8.** The quantitative analysis of H_2_O_2_ in HeLa cells treated with different nanoparticles. * denotes 0.01 < p < 0.05. **Fig. S9.** The quantitative analysis of SOD-1 and GPX4 expression in HeLa cells treated with different nanoparticles. * denotes 0.01 < p < 0.05 and ** denotes p < 0.01. **Fig. S10.** The protein expression of HeLa cells incubated with different samples. **Fig. S11.** (a) Representative photographs of mice, and (b) the excised solid tumors from the mice treated with different samples. **Fig. S12.** The relative expressive levels of cleaved caspase-3 in tumor tissues treated with different nanoparticles. * denotes 0.01 < p < 0.05, ** denotes p < 0.01, *** denotes p < 0.001 and **** denotes p < 0.0001. **Fig. S13.** The biodistribution of nanoparticles in mice treated with 10 mg/kg BMMP-Mn^2+^/Se/DOX via the tail vein. **Fig. S14.** The H&E staining of various organs in mice injected with different samples via the tail vein. Scale bar: 100 μm. **Table S1.** The cytotoxic effects of different samples on HeLa cells after incubation for 24 h.

## Data Availability

The datasets and materials used in the study are available from the corresponding author.

## References

[1] Yu L, Chen Y, Wu M, Cai X, Yu L, Chen Y, Wu M, Yao H, Zhang L, Chen H, Shi J (2016). "Manganese extraction” strategy enables tumor-sensitive biodegradability and theranostics of nanoparticles. J Am Chem Soc.

[2] Patra J, Das G, Fraceto L, Campos E, Torres M, Torres L, Torres L, Grillo R, Swamy M, Sharma S, Habtemariam S, Shin H (2018). Nano based drug delivery systems: recent developments and future prospects. J Nanobiotechnol.

[3] Wen J, Yang K, Liu F, Li H, Xu Y, Sun S (2017). Diverse gatekeepers for mesoporous silica nanoparticle based drug delivery systems. Chem Soc Rev.

[4] Zhang G, Du R, Qian J, Zheng X, Tian X, Cai D, He J, Wu Y, Huang W, Wang Y, Zhang X, Zhong K, Zou D, Wu Z (2018). A tailored nanosheet decorated with a metallized dendrimer for angiography and magnetic resonance imaging-guided combined chemotherapy. Nanoscale.

[5] Sun X, Zhang G, Du R, Xu R, Zhu D, Qian J, Bai G, Yang C, Zhang Z, Zhang X, Zou D, Wu Z (2019). A biodegradable MnSiO_3_@Fe_3_O_4_ nanoplatform for dual-mode magnetic resonance imaging guided combinatorial cancer therapy. Biomaterials.

[6] Xiao J, Xiao G, Xu R, Chen H, Tian G, Wang B, Yang C, Bai G, Zhang Z, Yang H, Zhong K, Zou D, Wu Z (2019). A pH-responsive platform combining chemodynamic therapy with limotherapy for simultaneous bioimaging and synergistic cancer therapy. Biomaterials.

[7] Ding C, Tong L, Feng J, Fu J (2016). Recent advances in stimuli-responsive release function drug delivery systems for tumor treatment. Molecules.

[8] Chen W, Zhou S, Ge L, Wu W, Jiang X (2018). Translatable high drug loading drug delivery systems based on biocompatible polymer nanocarriers. Biomacromolecules.

[9] Wang H, Dai T, Zhou S, Huang X, Li S, Sun K, Zhou G, Dou H (2017). Self-assembly assisted fabrication of dextran-based nanohydrogels with reduction-cleavable junctions for applications as efficient drug delivery systems. Sci Rep.

[10] Schrand A, Rahman M, Hussain S, Schlager J, Smith D, Syed A (2010). Metal-based nanoparticles and their toxicity assessment. Wiley Interdiscip Rev Nanomed Nanobiotechnol.

[11] He Y, Zeng B, Liang S, Long M, Xu H (2017). Synthesis of pH-responsive biodegradable mesoporous silica–calcium phosphate hybrid nanoparticles as a high potential drug carrier. ACS Appl Mater Interfaces.

[12] Bodewein L, Bodewein F, Fiore S, Hollert H, Fischer R, Fenske M (2016). Differences in toxicity of anionic and cationic PAMAM and PPI dendrimers in zebrafish embryos and cancer cell lines. Toxicol Appl Pharmacol.

[13] Chen Y, Hu X, Sun J, Zhou Q (2015). Specific nanotoxicity of graphene oxide during zebrafish embryogenesis. Nanotoxicology.

[14] Liu T, Wu S, Chen Y, Chou C, Chen C (2015). Biosafety evaluations of well-dispersed mesoporous silica nanoparticles: towards in vivo-relevant conditions. Nanoscale.

[15] Duan J, Yu Y, Li Y, Yu Y, Sun Z (2013). Cardiovascular toxicity evaluation of silica nanoparticles in endothelial cells and zebrafish model. Biomaterials.

[16] Jeong J, Cho H, Choi M, Lee W, Chung B, Lee J (2015). In vivo toxicity assessment of angiogenesis and the live distribution of nano-graphene oxide and its PEGylated derivatives using the developing zebrafish embryo. Carbon.

[17] Yuan Z, Li Y, Hu Y, You J, Higashisaka K, Nagano K, Tsutsumi Y, Gao J (2016). Chitosan nanoparticles and their Tween 80 modified counterparts disrupt the developmental profile of zebrafish embryos. Int J Pharm.

[18] Jia H, Zhu Y, Duan Q, Chen Z, Wu F (2019). Nanomaterials meet zebrafish: toxicity evaluation and drug delivery applications. J Control Release.

[19] Pitchaimani A, Nguyen T, Marasini R, Eliyapura A, Azizi T, Jaberi-Douraki M, Aryal S (2019). Biomimetic natural killer membrane camouflaged polymeric nanoparticle for targeted bioimaging. Adv Funct Mater.

[20] Rahman M, Ueda M, Hirose T, Ito Y (2018). Spontaneous formation of gating lipid domain in uniform-size peptide vesicles for controlled release. J Am Chem Soc.

[21] He H, Guo C, Wang J, Korzun W, Wang X, Ghosh S, Yang H (2018). Leutusome: a biomimetic nanoplatform integrating plasma membrane components of leukocytes and tumor cells for remarkably enhanced solid tumor homing. Nano Lett.

[22] Li S, Cheng H, Xie B, Qiu W, Zeng J, Li C, Wan S, Zhang L, Liu W, Zhang X (2017). Cancer cell membrane camouflaged cascade bioreactor for cancer targeted starvation and photodynamic therapy. ACS Nano.

[23] Deng G, Sun Z, Li S, Peng X, Li X, Zhou L, Ma Y, Gong P, Cai L (2018). Cell-membrane immunotherapy based on natural killer cell membrane coated nanoparticles for the effective inhibition of primary and abscopal tumor growth. ACS Nano.

[24] Ochyl L, Bazzill J, Charles P, Xu Y, Kuai R, Moon J (2018). PEGylated tumor cell membrane vesicles as a new vaccine platform for cancer immunotherapy. Biomaterials.

[25] Li S, Cheng H, Qiu W, Zhang L, Wan S, Zeng J, Zhang X (2017). Cancer cell membrane-coated biomimetic platform for tumor targeted photodynamic therapy and hypoxia-amplified bioreductive therapy. Biomaterials.

[26] Wang Z, An H, Hou D, Wang M, Zeng X, Zheng R, Wang L, Wang K, Wang H, Xu W (2019). Addressable peptide self-assembly on the cancer cell membrane for sensitizing chemotherapy of renal cell carcinoma. Adv Mater.

[27] Zhang K, Meng X, Yang Z, Cao Y, Cheng Y, Wang D, Lu H, Shi Z, Dong H, Zhang X (2019). Cancer cell membrane camouflaged nanoprobe for catalytic ratiometric photoacoustic imaging of microrna in living mice. Adv Mater.

[28] Zhang G, Gao J, Qian J, Zhang L, Zheng K, Zhong K, Cai D, Zhang X, Wu Z (2015). Hydroxylated mesoporous nanosilica coated by polyethylenimine coupled with gadolinium and folic acid: a tumor-targeted T_1_ magnetic resonance contrast agent and drug delivery system. ACS Appl Mater Interfaces.

[29] Xiao J, Zhang G, Qian J, Sun X, Tian J, Zhong K, Cai D, Wu Z (2018). Fabricating high-performance T_2_-weighted contrast agents via adjusting composition and size of nanomagnetic iron oxide. ACS Appl Mater Interfaces.

[30] Zhang G, Yang M, Cai D, Zheng K, Zhang X, Wu L, Wu Z (2014). Composite of functional mesoporous silica and DNA: an enzyme-responsive controlled release drug carrier system. ACS Appl Mater Interfaces.

[31] Yang P, Peng J, Chu Z, Jiang D, Jin W (2017). Facile synthesis of Prussian blue nanocubes/silver nanowires network as a water-based ink for the direct screen-printed flexible biosensor chips. Biosens Bioelectron.

[32] He Q, Gao Y, Zhang L, Zhang Z, Gao F, Ji X, Li Y, Shi J (2011). A pH-responsive mesoporous silica nanoparticles-based multi-drug delivery system for overcoming multi-drug resistance. Biomaterials.

[33] Tian G, Zheng X, Zhang X, Yin W, Yu J, Wang D, Zhang Z, Yang X, Gu Z, Zhao Y (2015). TPGS-stabilized NaYbF_4_:Er upconversion nanoparticles for dual-modal fluorescent/CT imaging and anticancer drug delivery to overcome multi-drug resistance. Biomaterials.

[34] Zhu H, Chen H, Zeng X, Wang Z, Zhang X, Wu Y, Gao Y, Zhang J, Liu K, Liu R, Cai L, Mei L, Feng S (2014). Co-delivery of chemotherapeutic drugs with vitamin E TPGS by porous PLGA nanoparticles for enhanced chemotherapy against multi-drug resistance. Biomaterials.

[35] Lehouritis P, Stanton M, McCarthy F, Jeavons M, Tangney M (2016). Activation of multiple chemotherapeutic prodrugs by the natural enzymolome of tumour-localised probiotic bacteria. J Control Release.

[36] Pan Q, Chen T, Nie C, Yi J, Liu C, Hu Y, Chu X (2018). In situ synthesis of ultrathin ZIF-8 film coated MSNs for co-delivering Bcl-2 siRNA and doxorubicin to enhance chemotherapeutic efficacy in drug-resistant cancer cells. ACS Appl Mater Interfaces.

[37] Chang L, Guo R (2017). Comparison of the efficacy among multiple chemotherapeutic interventions combined with radiation therapy for patients with cervix cancer after surgery: a network meta-analysis. Oncotarget.

[38] Liu Y, Jiang Y, Zhang M, Tang Z, He M, Bu W (2018). Modulating hypoxia via nanomaterials chemistry for efficient treatment of solid tumors. Acc Chem Res.

[39] Zhang C, Bu W, Ni D, Zhang S, Li Q, Yao Z, Zhang J, Yao H, Wang Z, Shi J (2016). Synthesis of iron nanometallic glasses and their application in cancer therapy by a localized Fenton reaction. Angew Chem Int Ed.

[40] Dai Y, Cheng S, Wang Z, Zhang R, Yang Z, Wang J, Yung B, Wang Z, Jacobson O, Xu C, Ni Q, Yu G, Zhou Z, Chen X (2018). Hypochlorous acid promoted platinum drug chemotherapy by myeloperoxidase-encapsulated therapeutic metal phenolic nanoparticles. ACS Nano.

[41] Yang Z, Dai Y, Yin C, Fan Q, Zhang W, Song J, Yu G, Tang W, Fan W, Yung B, Li J, Li X, Li X, Tang Y, Huang W, Song J, Chen J (2018). Activatable semiconducting theranostics: simultaneous generation and ratiometric photoacoustic imaging of reactive oxygen species in vivo. Adv Mater.

[42] Tang Z, Zhang H, Liu Y, Ni D, Zhang H, Zhang J, Yao Z, He M, Shi J, Bu W (2017). Antiferromagnetic pyrite as the tumor microenvironment-mediated nanoplatform for self-enhanced tumor imaging and therapy. Adv Mater.

[43] Zhang G, Zhang L, Si Y, Li Q, Xiao J, Wang B, Liang C, Wu Z, Tian G (2020). Oxygen-enriched Fe_3_O_4_ /Gd_2_O_3_ nanopeanuts for tumor-targeting MRI and ROS-triggered dual-modal cancer therapy through platinum (IV) prodrugs delivery. Chem Eng J.

[44] Wang H, Gao Z, Liu X, Agarwal P, Zhao S, Conroy D, Ji G, Yu J, Jaroniec C, Liu Z, Lu X, Li X, He X (2018). Targeted production of reactive oxygen species in mitochondria to overcome cancer drug resistance. Nat Commun.

[45] Sarin L, Sanchez V, Yan A, Kane A, Hurt R (2010). Selenium-carbon bifunctional nanoparticles for the treatment of malignant mesothelioma. Adv Mater.

[46] Liu Y, Zhao J, Zhang Y, Zhang H, Zhang Z, Gao H, Mao Y (2019). Enhanced single-band red upconversion luminescence of α-NaErF4: Mn nanoparticles by a novel hollow-shell structure under multiple wavelength excitation. J Alloy Compd.

[47] Lin L, Huang T, Song J, Ou X, Wang Z, Deng H, Tian R, Liu Y, Wang J, Liu Y, Yu G, Zhou Z, Wang S, Niu G, Yang H, Chen X (2019). Synthesis of copper peroxide nanodots for H_2_O_2_ self-supplying chemodynamic therapy. J Am Chem Soc.

[48] Shi J, Su Y, Liu W, Chang J, Zhang Z (2017). A nanoliposome-based photoactivable drug delivery system for enhanced cancer therapy and overcoming treatment resistance. Int J Nanomed.

[49] Chen Y, Yao Y, Zhou X, Liao C, Dai X, Liu J, Yu Y, Zhang S (2019). Cascade-reaction-based nanodrug for combined chemo/starvation/chemodynamic therapy against multidrug-resistant tumors. ACS Appl Mater Interfaces.

[50] Chen T, Hou P, Zhang Y, Ao R, Su L, Jiang Y, Zhang Y, Cai H, Wang J, Chen Q, Song J, Lin L, Yang H, Chen X (2021). Singlet oxygen generation in dark-hypoxia by catalytic microenvironment-tailored nanoreactors for NIR-II fluorescence-monitored chemodynamic therapy. Angew Chem Int Ed.

[51] Lin L, Song J, Song L, Ke K, Liu Y, Zhou Z, Shen Z, Li J, Yang Z, Tang W, Niu G, Yang H, Chen X (2018). Simultaneous Fenton-like ion delivery and glutathione depletion by MnO_2_-based nanoagent to enhance chemodynamic therapy. Angew Chem Int Ed.

