# Supplementary material for: A nanoselenium-coating biomimetic cytomembrane nanoplatform for mitochondrial targeted chemotherapy- and chemodynamic therapy through manganese and doxorubicin codelivery
Source: J Nanobiotechnology. 2021 Jul 30;19:227. doi: 10.1186/s12951-021-00971-9 (PMC8325191; doi:10.1186/s12951-021-00971-9)
Supplement: Supplementary file 1 — Additional file 1: Fig. S1. The EDX spectrum of BMMP-Mn2+/Se. Fig. S2. The UV absorption spectra of DOX before and after loading. Fig. S3. (a) The color changes of the mixture of BMMP-Mn2+/Se in different pH solutions and (b) the different concentration of BMMP-Mn2+/Se at pH 5.5 solution. Fig. S3. The UV absorption spectra of different nanoparticles mixed with TMB, and H2O2 in solution. Fig. S4. (a) The flow cytometry analysis of HeLa cells treated with FITC-labeled BMMP-Mn2+/Se/DOX at different concentration. (b) The flow cytometry analysis HeLa cells treated with FITC-labeled BMMP-Mn2+/Se/DOX for different time. (c) The flow cytometry analysis for endocytosis pathway of BMMP-Mn2+/Se/DOX nanoparticles. (d) The CLSM images of HeLa cells treated with FITC-labeled BMMP-Mn2+/Se/DOX for different time, the same scale bar applies to all images. Scale bar: 20 μm. Fig. S5. The flow cytometry analysis of intracellular ROS in HeLa cells. Fig. S6. The morphology of HGF cells treated with different concentrations of BMMP-Mn2+ for 24 h. Scale bar: 100 μm. Fig. S7. The viability of (a) MCF-7 and (b) MCF-7/ADR cells treated with free DOX and BMMP-Mn2+/Se/DOX for 24 h. Fig. S8. The quantitative analysis of H2O2 in HeLa cells treated with different nanoparticles. * denotes 0.01 < p < 0.05. Fig. S9. The quantitative analysis of SOD-1 and GPX4 expression in HeLa cells treated with different nanoparticles. * denotes 0.01 < p < 0.05 and ** denotes p < 0.01. Fig. S10. The protein expression of HeLa cells incubated with different samples. Fig. S11. (a) Representative photographs of mice, and (b) the excised solid tumors from the mice treated with different samples. Fig. S12. The relative expressive levels of cleaved caspase-3 in tumor tissues treated with different nanoparticles. * denotes 0.01 < p < 0.05, ** denotes p < 0.01, *** denotes p < 0.001 and **** denotes p < 0.0001. Fig. S13. The biodistribution of nanoparticles in mice treated with 10 mg/kg BMMP-Mn2+/Se/DOX via the tail vein. [file 12951_2021_971_MOESM1_ESM.docx]

**Supporting Information**

**A Nanoselenium-coating Biomimetic Cytomembrane Nanoplatform for Mitochondrial Targeted Chemotherapy**- **and Chemodynamic Therapy through Manganese and Doxorubicin Codelivery**

Jianmin Xiao^1,2†^, Miao Yan^1,2†^, Ke Zhou^5†^, Hui Chen^4^, Zhaowei Xu^3^, Yuehao Gan^1,2^, Biao Hong^1,2^, Geng Tian^3^, Junchao Qian^5^*, Guilong Zhang^3^*, Zhengyan Wu^1^*

^1^Key Laboratory of High Magnetic Field and Ion Beam Physical Biology, Hefei Institutes of Physical Science, Chinese Academy of Sciences, Hefei 230031, P.R. China.

^2^University of Science and Technology of China, Hefei 230026, P.R. China

^3^School of Pharmacy, the Key Laboratory of Prescription Effect and Clinical Evaluation of State Administration of Traditional Chinese Medicine of China, Binzhou Medical University, Yantai 264003, P.R. China

^4^Department of Dental Implant Center, Stomatologic Hospital & College, Anhui Medical University, Key Laboratory of Oral Diseases Research of Anhui Province, Hefei 230032, P.R. China

^5^Hefei Cancer Hospital, Anhui Province Key Laboratory of Medical Physics and Technology, Institute of Health and Medical Technology, Hefei Institutes of Physical Science, Chinese Academy of Sciences, Hefei 230031, P.R. China

∗Correspondence: [qianjunchao@hmfl.ac.cn](mailto:qianjunchao@hmfl.ac.cn), [glzhang@bzmc.edu.cn](mailto:glzhang@bzmc.edu.cn), [zywu@ipp.ac.cn](mailto:zywu@ipp.ac.cn).

^†^Jianmin Xiao, Miao Yan, and Ke Zhou contributed equally to this work


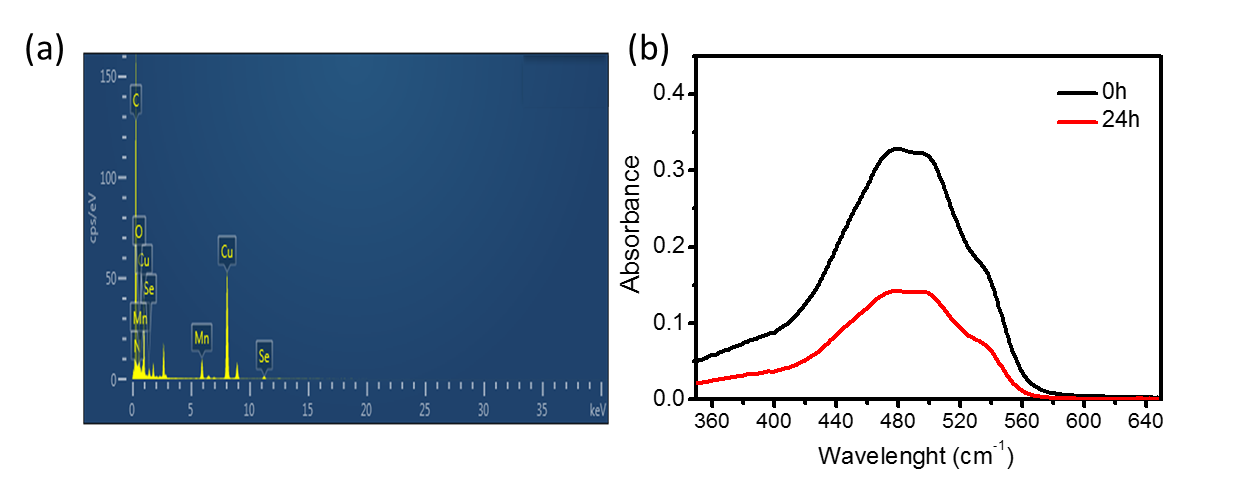


Fig. S1 (a) The EDX spectrum of BMMP-Mn^2+^/Se.

Fig. S2 The UV absorption spectra of DOX before and after loading.


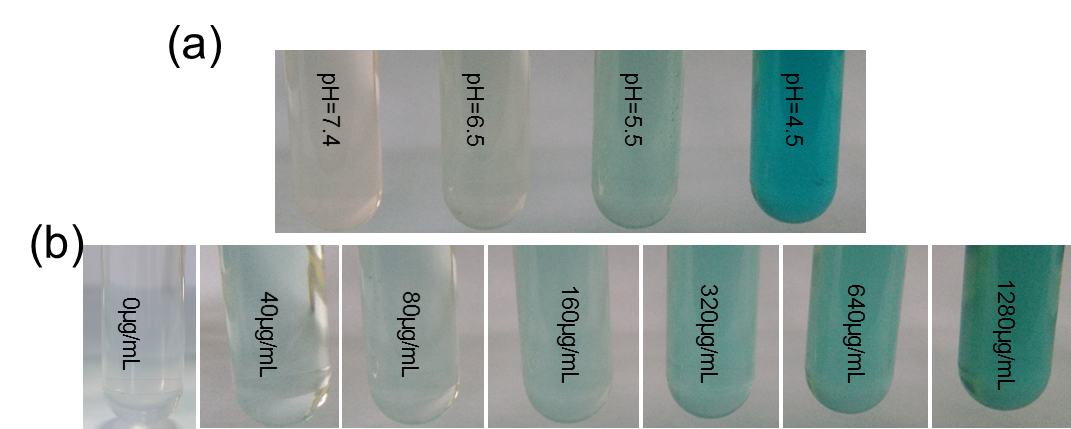


Fig. S3 (a) The color changes of the mixture of BMMP-Mn^2+^/Se in different pH solutions and (b) the different concentration of BMMP-Mn^2+^/Se at pH 5.5 solution.


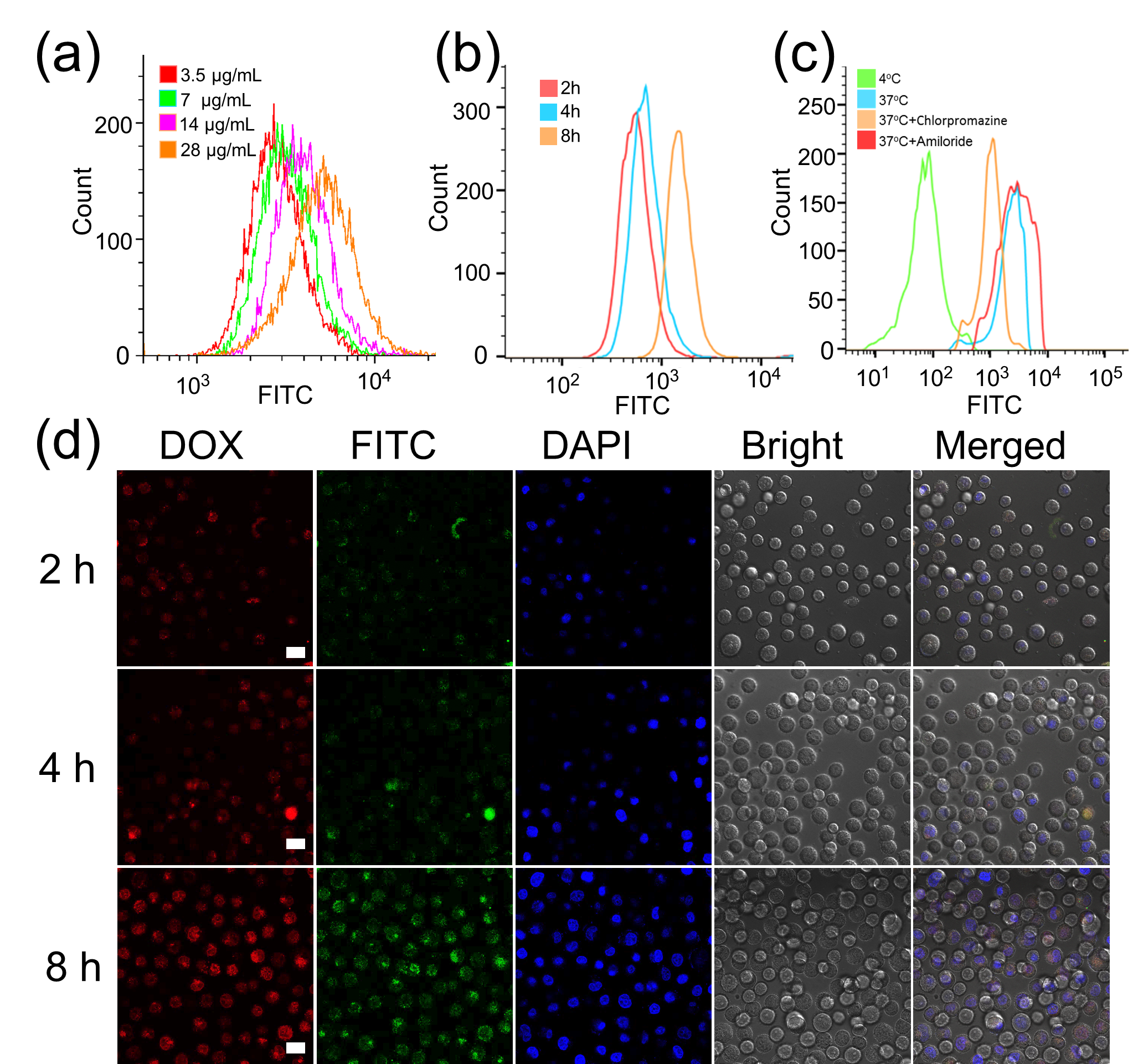


Fig. S4 (a) The flow cytometry analysis of HeLa cells treated with FITC-labeled BMMP-Mn^2+^/Se/DOX at different concentration. (b) The flow cytometry analysis of HeLa cells treated with FITC-labeled BMMP-Mn^2+^/Se/DOX for different time. (c) The flow cytometry analysis for endocytosis pathway of BMMP-Mn^2+^/Se/DOX nanoparticles. (d) The CLSM images of HeLa cells treated with FITC-labeled BMMP-Mn^2+^/Se/DOX for different time, the same scale bar applies to all images. Scale bar: 20 μm.


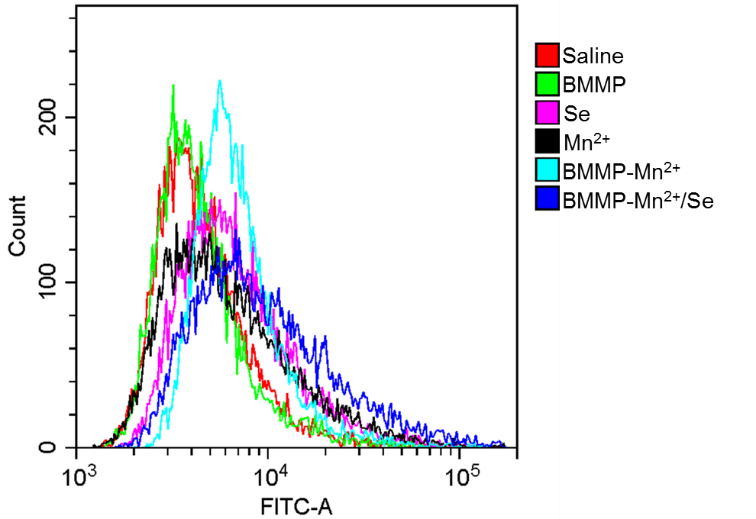


Fig. S5 The flow cytometry analysis of intracellular ROS in HeLa cells.


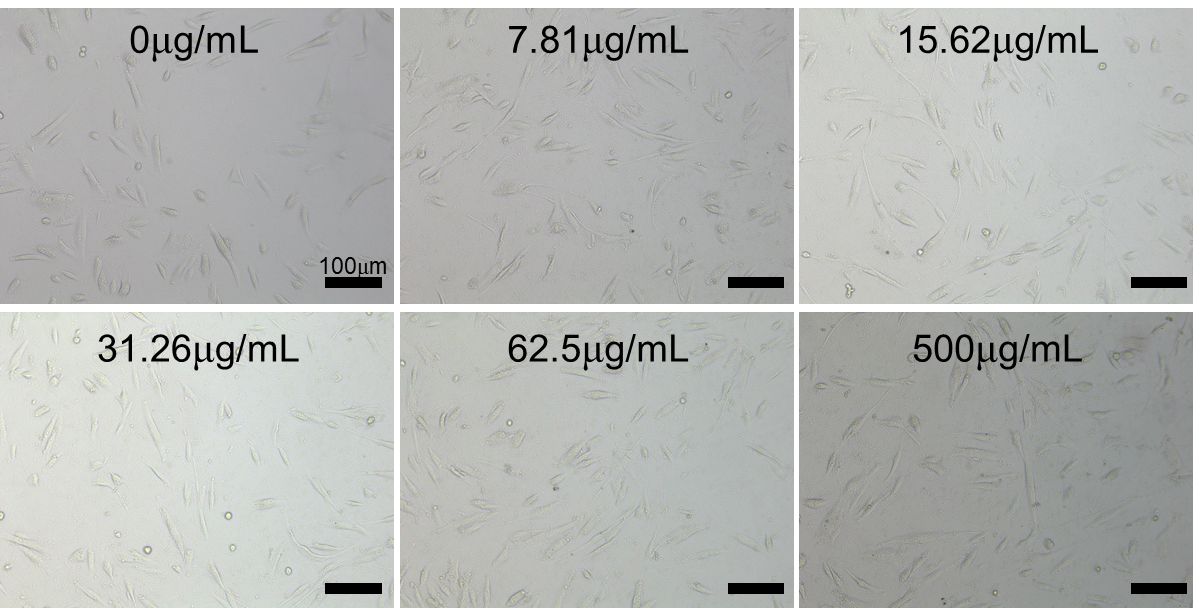


Fig. S6 The morphology of HGF cells treated with different concentrations of BMMP-Mn^2+^ for 24 h. Scale bar: 100 μm.


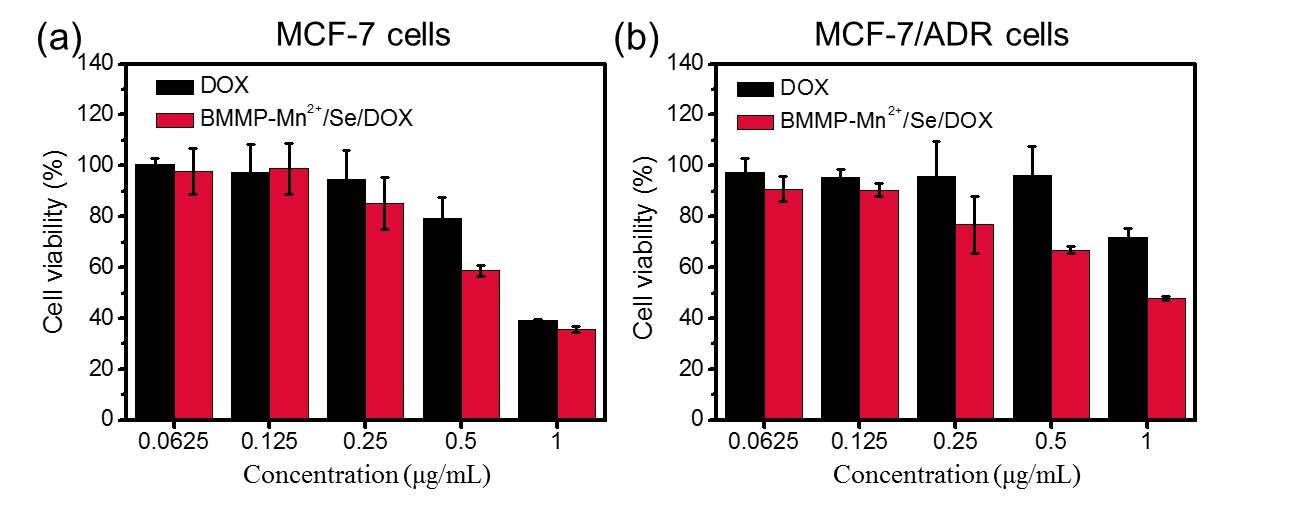


Fig. S7 The viability of (a) MCF-7 and (b) MCF-7/ADR cells treated with free DOX and BMMP-Mn^2+^/Se/DOX for 24 h.


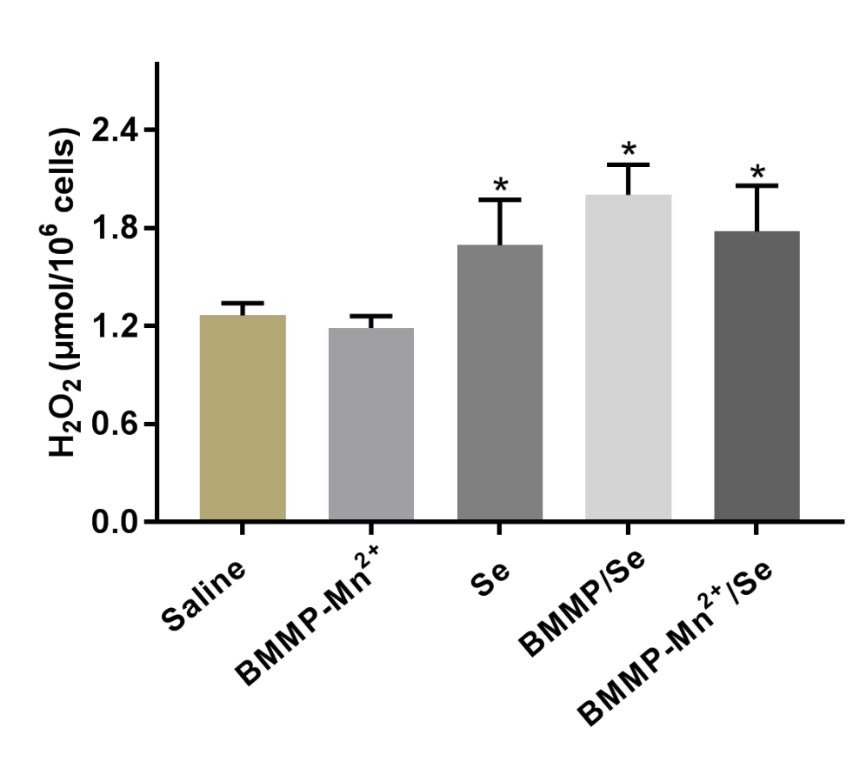


Fig. S8 The quantitative analysis of H_2_O_2_ in HeLa cells treated with different nanoparticles. * denotes 0.01 < p < 0.05.


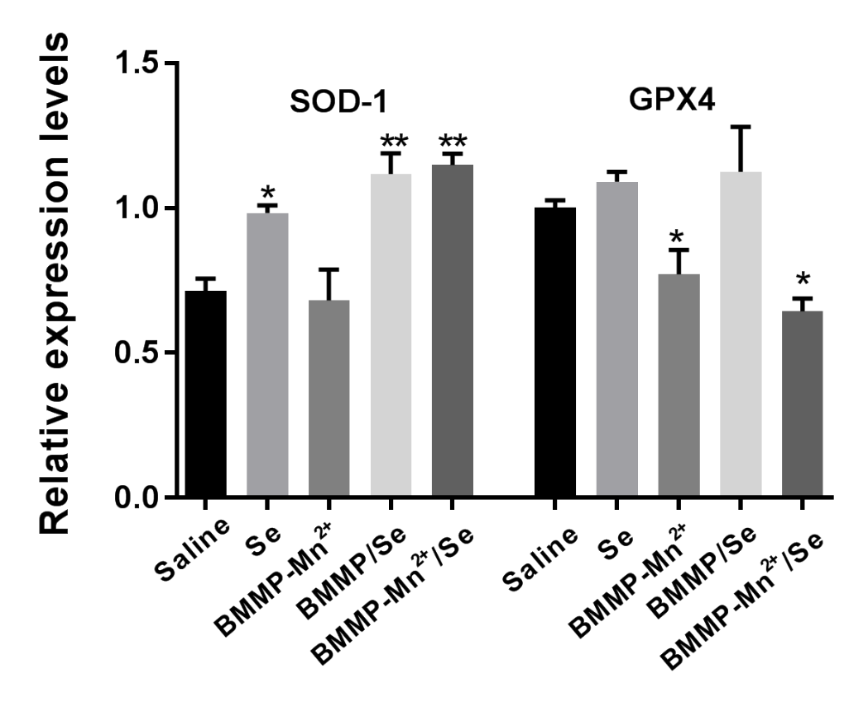


Fig. S9 The quantitative analysis of SOD-1 and GPX4 expression in HeLa cells treated with different nanoparticles. * denotes 0.01 < p < 0.05 and ** denotes p < 0.01.


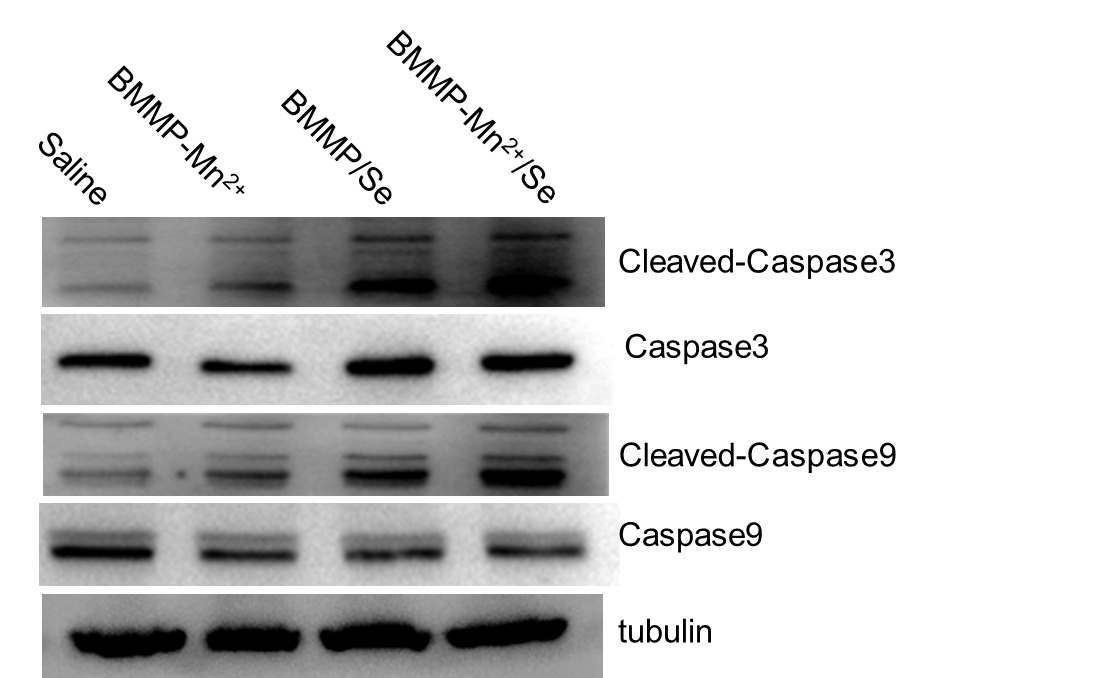


Fig. S10 The protein expression of HeLa cells incubated with different samples.


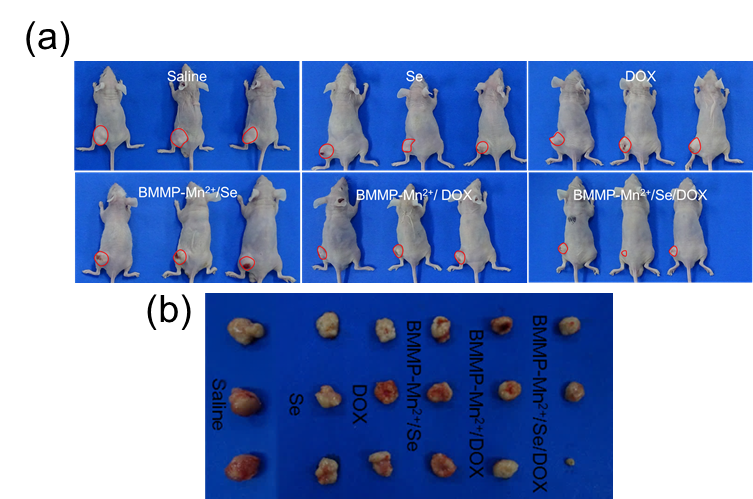


Fig. S11 (a) Representative photographs of mice, and (b) the excised solid tumors from the mice treated with different samples.


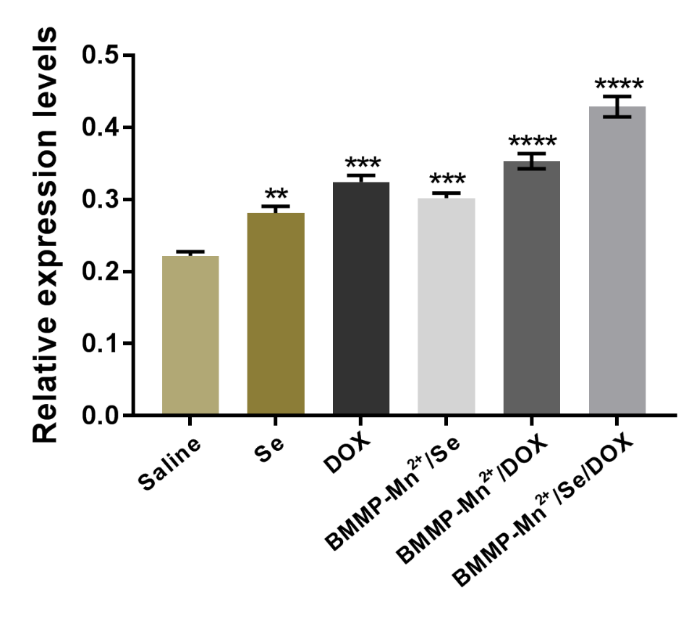


Fig. S12 The relative expressive levels of cleaved caspase-3 in tumor tissues treated with different nanoparticles. * denotes 0.01 < p < 0.05, ** denotes p < 0.01, *** denotes p < 0.001 and **** denotes p < 0.0001.


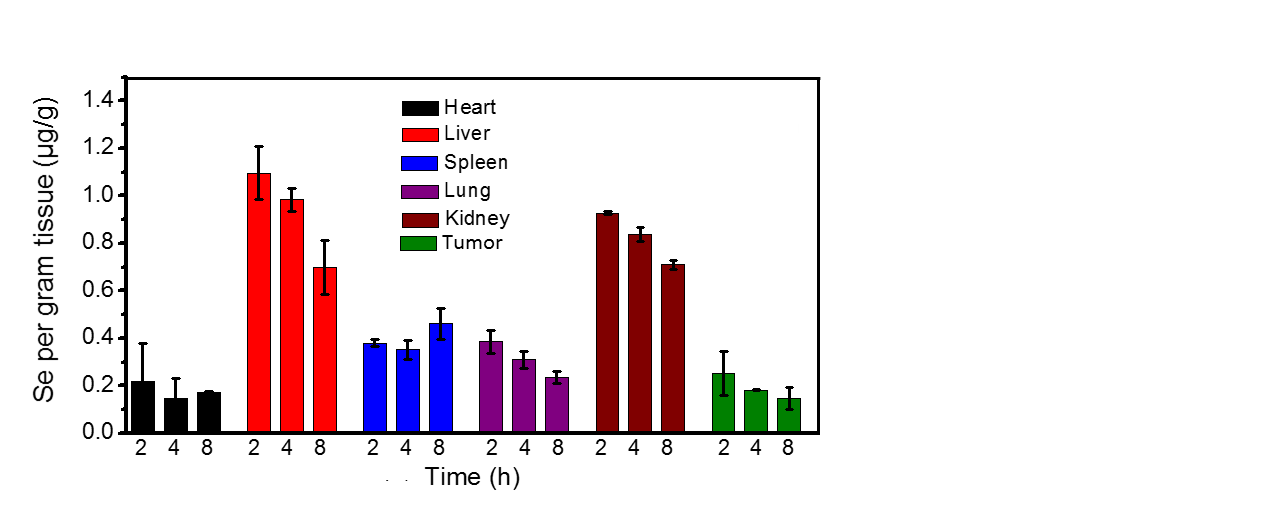


Fig. S13 The biodistribution of nanoparticles in mice treated with 10 mg/kg BMMP-Mn^2+^/Se/DOX via the tail vein.


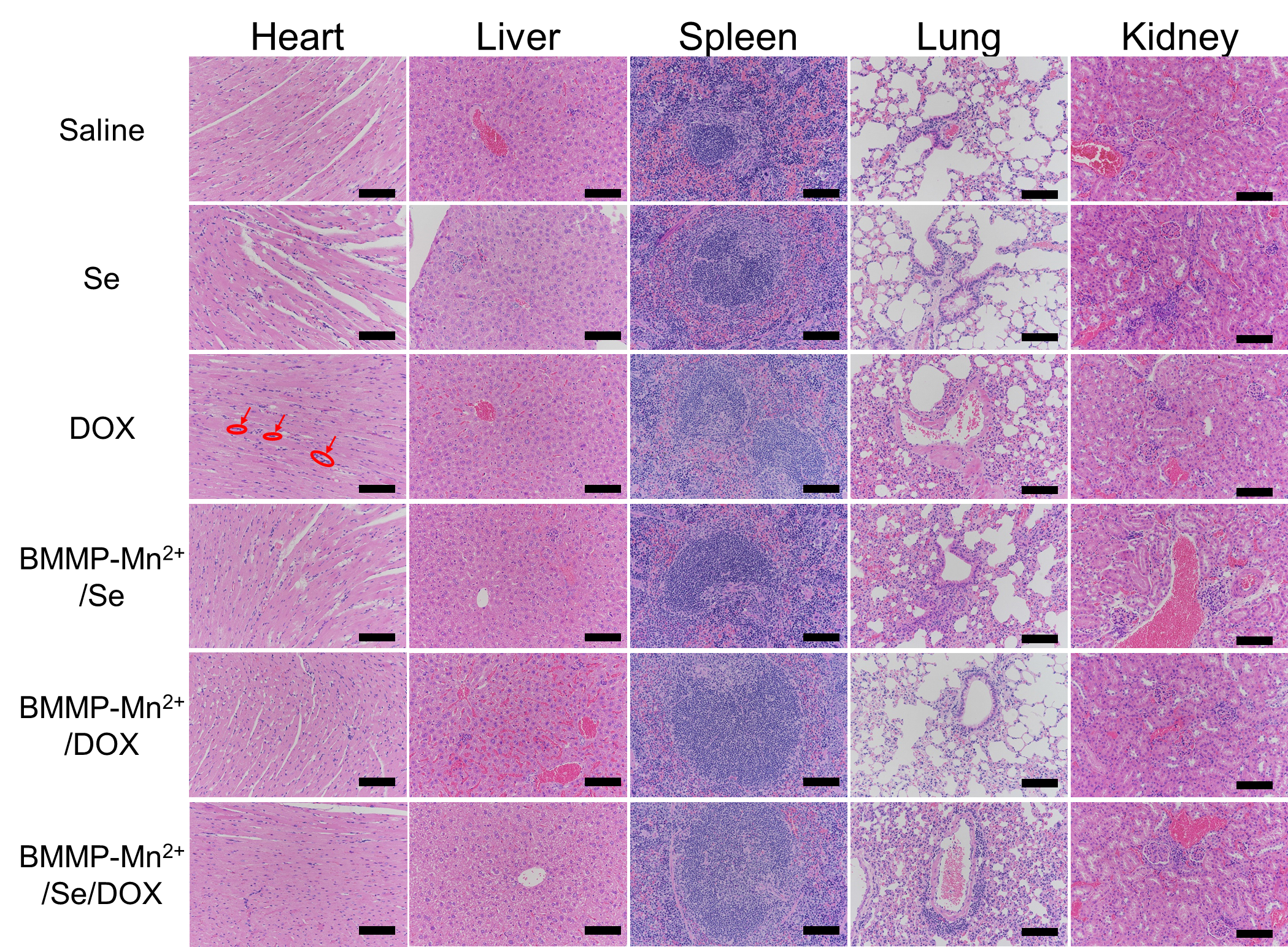


Fig. S14 The H&E staining of various organs in mice injected with different samples via the tail vein. Scale bar: 100 μm.

Table S1 The cytotoxic effects of different samples on HeLa cells after incubation for 24 h.

| samples | IC_50_ (ug/mL) |
| --- | --- |
| Se | 42.9 |
| DOX | 1.6 |
| BMMP-Mn^2+^/Se | 2.1 |
| BMMP-Mn^2+^/DOX | 1.5 |
| BMMP-Mn^2+^/Se/DOX | 1.2 |
